# Click Triazole as a Linker for Pretargeting Strategies: Synthesis, Docking Investigations, Fluorescence Diagnosis, and Antibacterial Action Studies

**DOI:** 10.3390/molecules28062758

**Published:** 2023-03-18

**Authors:** Qian Liu, Mingxia Zhao, Cairong Song, Jiankang Sun, Jiali Tao, Bin Sun, Junbing Jiang

**Affiliations:** 1 Department of Veterinary Medicine, Shanxi Agricultural University, Jinzhong 030801, China; 2 Department of Mining Engineering, Shanxi Institute of Engineering and Technology, Yangquan 045000, China

**Keywords:** 1,2,3-triazoles, derivative, click reaction, antibacterial activity, fluorescence detection

## Abstract

In this study, three compounds **A1**, **A2,** and **A3** and fluorescent probes **T1**, **T2**, **T3**, and **T4** were designed and synthesized. ^1^H NMR, ^13^C NMR, and MS characterization and elemental analysis were used to confirm **A1**–**A3** and **T1**–**T4**. **A1**–**A3** and **T1**–**T4** formed diagnostic molecules by “click” reactions. **A1**–**A3** and **T1**–**T4** did not significantly increase cell death at concentrations of 80 μmol/L. Preliminary screening of the compounds for antibacterial activity revealed that **A2** has better antibacterial activity against *Agrobacterium tumefaciens*. The synthesized compounds and fluorescent probes can be targeted and combined in the physiological condition to form diagnostic molecules for fluorescence detection of *Agrobacterium tumefaciens*. The binding sites of **A1**–**A3** were deduced theoretically using the AutoDock Vina software docking tool. Further study of the mechanism of the antibacterial action of these compounds is likely to identify new agents against resistant bacterial strains.

## 1. Introduction

Diagnostic technology has always been an important direction in the research of pathogenic diseases [1,2,3,4]. With the development of molecular biology, some common pathogens have been quickly and accurately diagnosed [5,6]. At present, the diagnostic methods of pathogenic bacteria can be roughly divided into four categories: pathogenic diagnostic methods, serological diagnosis, molecular biological diagnosis, and immunological diagnosis, and detection methods that use organic small molecule fluorescent probes to target specific proteins of pathogenic bacteria through biological orthogonal targeting [7,8,9,10,11,12].

Most of the methods of detecting pathogens with organic small molecule fluorescent probes use their bioorthogonal reactions to target specific virulence proteins of pathogens or design and synthesize specific fluorescent probes according to the microenvironment of pathogens [9,10,13,14]. Usually, the “click” reaction in click chemistry is used to modify the target protein by bioorthogonal method to achieve the “click” reaction combined with the fluorescent probe in the physiological condition in order to achieve pathogen detection [15,16]. During the past decades, the seminal work on “click chemistry” by Huisgen, followed by further independent development by Meldal et al. and Sharpless-Fokin [17,18,19,20], has significantly impacted the fields of chemical biology, sensing and diagnosis, materials chemistry, drug discovery, and drug delivery [21,22,23].

Bioorthogonal chemistry realizes the pretargeting approach [24,25,26,27,28]. B8I-2 was discovered by Smith Mark A. et al. to be able to attach to VirB8’s surface groove close to the dimerization site and prevent the assembly of the T4SS system [29,30,31,32,33]. According to B8I-2, we designed and synthesized three compounds **A1**, **A2,** and **A3** targeting VirB8 and **A1**, **A2,** and **A3** “click” with the four fluorescent probes **T1**, **T2**, **T3,** and **T4**. In this study, we use pretargeting strategies to form an integrated diagnostic molecule for targeted fluorescence detection of *Agrobacterium tumefaciens*.

## 2. Results and Discussion

### 2.1. Design and Synthesis of B8I-2 Derivatives and Fluorescent Probes

**Figure 1:**
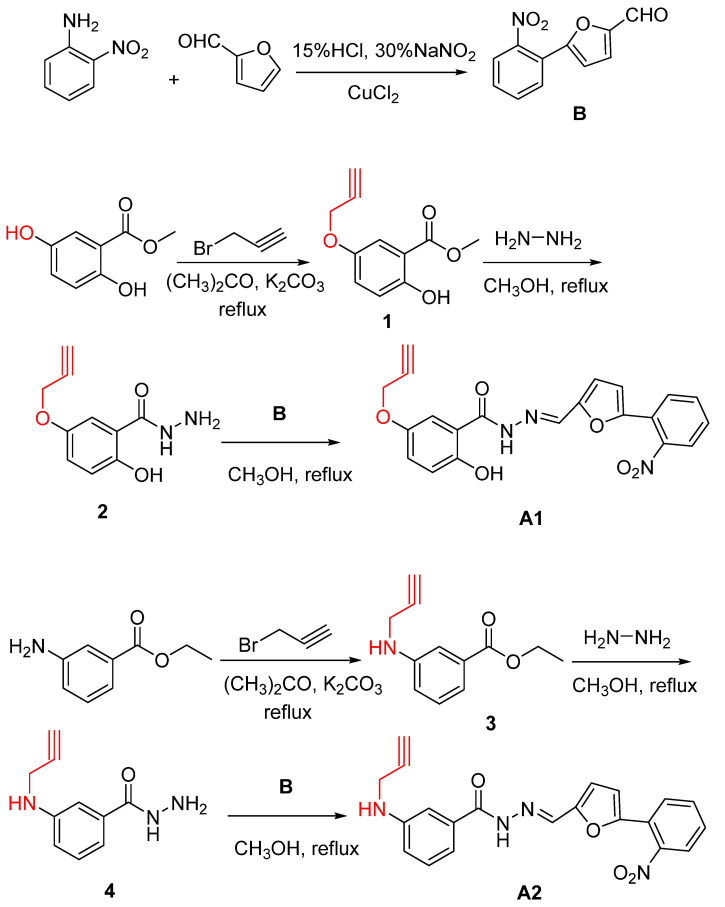

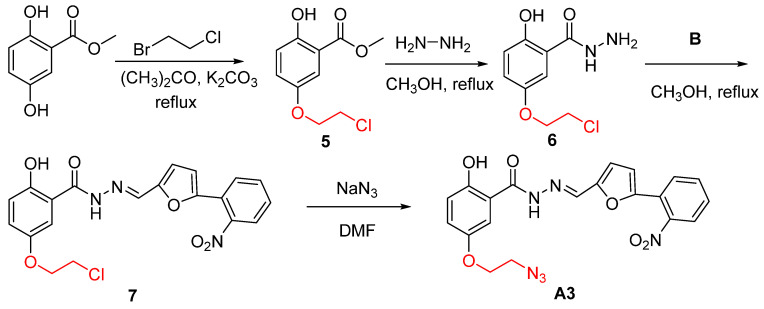
Synthesis routes of compounds **A1**–**A3**.

According to the literature [34,35,36,37], the compounds **A1**, **A2,** and **A3** were designed (Figure 1). Using Methyl 2,5-dihydroxybenzoate and Ethyl 3-aminobenzoate as the starting material, the reaction with 3-bromopropyne and 1,2-Dibromoethane eliminated a hydrogen bromide molecule, and then reacted with hydrazine hydrate to produce a compound containing N-N-hydrazide, and finally combined with o-nitroaniline and furfural products. The compounds **A1**, **A2,** and **A3** targeting the T4SS were synthesized. The structures of **A1**–**A3** were confirmed by ^1^H NMR, ^13^C NMR, and MS characterization and elemental analysis (Figures S1–S3 in the Supplementary Materials).

**Figure 2:**
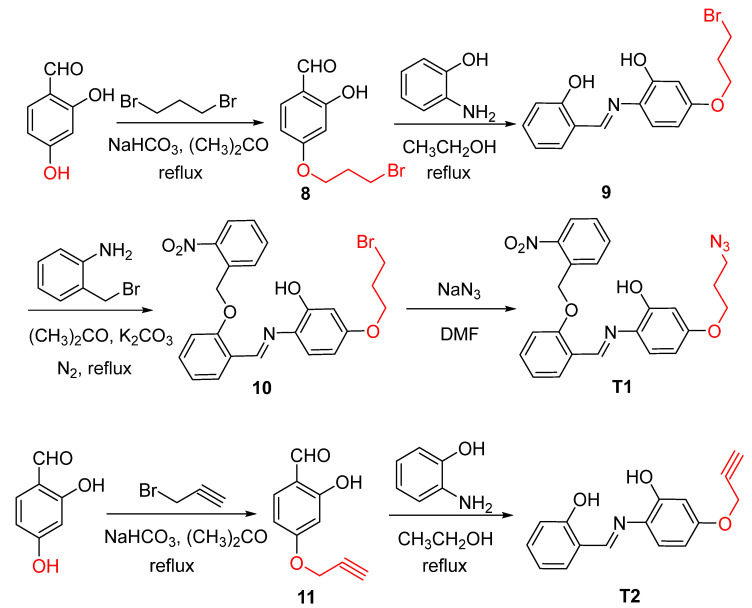

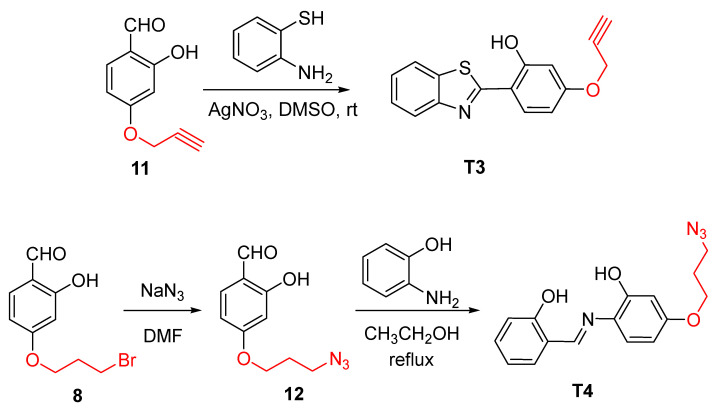
Synthesis routes of the probes **T1**–**T4**.

According to the literature [38,39,40,41,42,43,44], 2,5-Dihydroxybenzaldehyde is used as the raw material, and the alkyne group or azide is connected to the hydroxyl position at the fifth position (Figure 2). Then, the probes **T1**, **T2**, **T3**, and **T4** containing alkyne groups or azide groups are obtained by reacting with 2-Aminophenthiol and 2-Aminothiophenol, respectively. The structures of **T1**–**T4** were confirmed by ^1^H NMR, ^13^C NMR, and MS characterization and elemental analysis (Figures S4–S7 in the Supplementary Materials).

### 2.2. UV-Visible Absorption Spectroscopy and Fluorescence Spectroscopic

**Figure 3:**
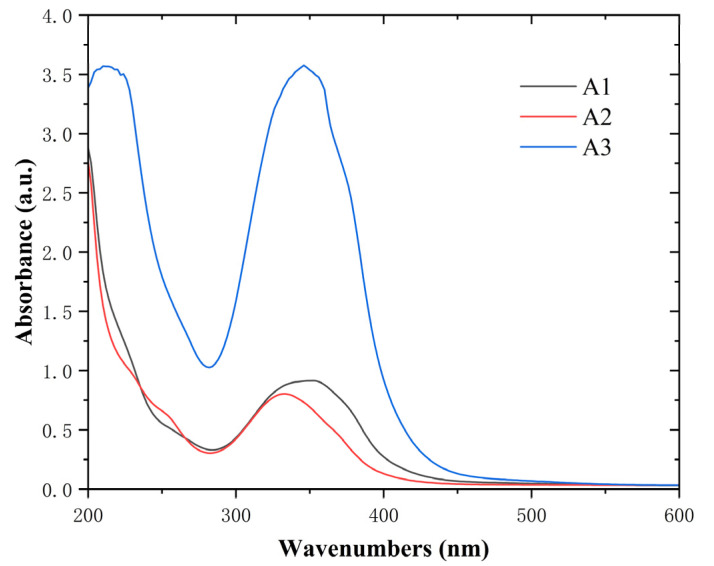
Experimental absorption spectra of **A1** (black), **A2** (red), and **A3** (blue).

To obtain the optical properties, UV-Vis analyses of **A1**, **A2,** and **A3** were carried out. As shown in the UV–Vis absorption spectrum (Figure 3), the UV-Vis spectra for compounds **A1**–**A3** were measured in methanol. The absorption maxima (λ_max_) for **A1**, **A2,** and **A3** lie at 352 nm, 332 nm, and 346 nm. The absorption spectra of **A1**–**A3** are almost identical.

**Figure 4:**
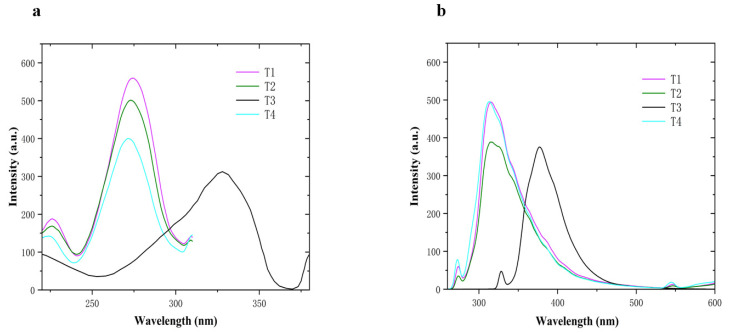
(**a**) The excitation wavelengths of **T1**–**T4** (**b**). The emission wavelengths of **T1**–**T4**.

The excitation wavelength and emission wavelength are essential parameters for fluorescence detection. Choosing the appropriate excitation and emission wavelengths is important for both sensitivity and selectivity of detection. The best excitation wavelength of **T1**, **T2**, **T3**, and **T4** is 273, 273, 327, and 272 nm, respectively, which corresponds to Figure 4a. The best emission wavelength of **T1**, **T2**, **T3**, and **T4** is 310, 315, 377, and 313 nm, respectively, which corresponds to Figure 4b.

### 2.3. The HPLC-MS Results for the “Click” Reactions

The HPLC-MS results of the “click” reactions are shown in the Supplementary Materials, and the results of click 1 are shown in Figure S8. The results of click 2 are shown in Figure S9 and the results of click 3 are shown in Figure S10. Based on the HPLC-MS results, the optimal reaction time of the three “click” reactants were determined to be 60 min and the optimal reaction ratio was 1:1.

### 2.4. Determination of the Maximum Safe Concentration of the Drugs

**Figure 5:**
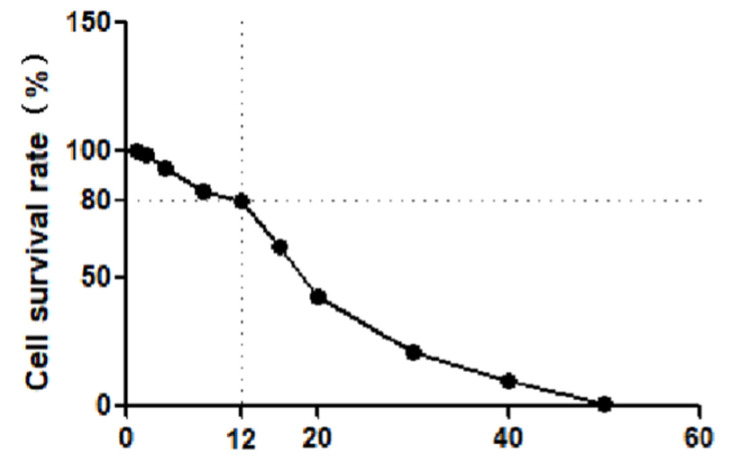
Effects of different concentrations of copper sulfate solution on the survival rate of RAW264.7 cell.

The test results are shown in Figure 5. The cell survival rate was 80% when the copper sulfate concentration was 12 µmol/L. The IC_50_ values of **A1**, **A2**, **A3**, **T1**, **T2**, **T3**, and **T4** on RAW264.7 cell are shown in Table 1. The IC_50_ values of the seven compounds on RAW264.7 cell were all above 80 µmol/L, indicating that they had no obvious toxicity to normal cells at 80 µmol/L. The cytotoxicity test indicated the safety and reliability of the obtained compounds as chemical diagnostic drugs to a certain extent and laid a foundation for subsequent experiments.

### 2.5. Antibacterial Activity on Agrobacterium Tumefaciens

**Figure 6:**
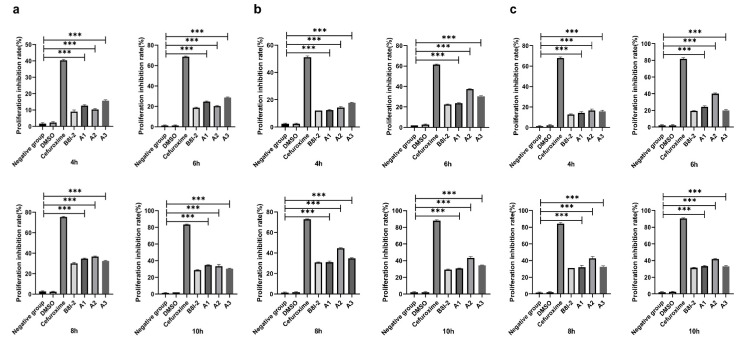
Antibacterial activity rate (%) of **A1**–**A3** treatment: (**a**) the antibacterial activity of the **A1**–**A3** on *Agrobacterium tumefaciens* with different times at 30 µmol/L; (**b**) the antibacterial activity of the **A1**–**A3** on *Agrobacterium tumefaciens* with different times at 50 µmol/L; and (**c**) the antibacterial activity of the **A1**–**A3** on *Agrobacterium tumefaciens* with different times at 100 µmol/L. Data represent the mean ± standard deviation (SD) of three independent experiments; *** represents *p* < 0.001.

The antibacterial screening of **A1**–**A3** was evaluated by lysogeny broth (LB) microdilution assays. The results indicated an antibacterial effect exhibited by **A1**–**A3** against *Agrobacterium tumefaciens*. To judge the influence of antibacterial activity and reaction time on bacteriostatic effect, the data were obtained after exposure for 4, 6, 8, and 10 h at concentrations of 30, 50, and 100 µmol/L (Figure 6). There were significant differences between **A1**–**A3** and the negative control group (*p* < 0.001). Among them, all groups showed the highest difference at 8 h of culture, indicating that the antibacterial effect of the three inhibitors was relatively good at 8 h. After 8 h, the antibacterial rates of **A1**–**A3** tended to be stable or decreased. The antibacterial activity of **A2** and **A3** at a concentration of 50 µmol/L was relatively good when cultured for 8 h. The antibacterial activities of **A2** and **A3** were 45% and 35%, respectively. The antibacterial activity of **A1** was the best when cultured for 8 h at a concentration of 30 µmol/L, and the antibacterial activity was 35%. In general, **A2** had a better antibacterial activity than **A1** and **A3**. From the overall trend of antibacterial activity, the antibacterial activity time of the **A1**–**A3** was 4–10 h.

### 2.6. Fluorescence Detection of Agrobacterium tumefaciens by Diagnostic Molecules

**Figure 7:**
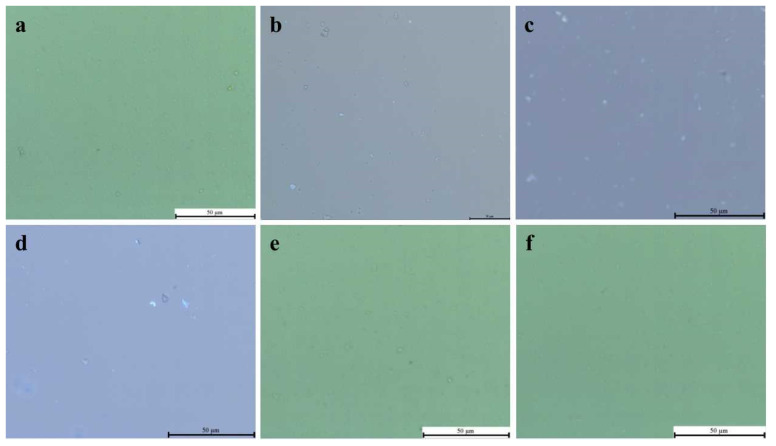
Diagnostic molecular targeted *Agrobacterium tumefaciens* fluorescence assay: (**a**) fluorescence detection of *Agrobacterium tumefaciens* without inhibitor and probe; (**b**) agrobacterium fluoresce was detected by adding inhibitor A1 and probe T4; (**c**) fluorescence detection of Agrobacterium with inhibitor A2 and probe T4; (**d**) agrobacterium fluorescence detection with inhibitor A3 and probe T3; (**e**) only Agrobacterium tumefacien probe T4 was added for fluorescence detection; (**f**) only Agrobacterium tumefacien probe T3 was added for fluorescence detection.

According to the results (Figure 7), the synthesized compound and fluorescent probe can be targeted and combined in the physiological condition to form diagnostic molecules for fluorescence detection of *Agrobacterium tumefaciens*. The blank group did not emit fluorescence, and the influence of *Agrobacterium tumefaciens* itself on the test was eliminated (Figure 7a). The results show that the diagnostic molecules formed by **A1** and **T4** emit fluorescence targeting *Agrobacterium tumefaciens*, but the fluorescence intensity is not strong; there is only weak fluorescence (Figure 7b). The diagnostic molecule formed by **A2** and **T4** had a better effect in targeting the fluorescence of *Agrobacterium tumefaciens* (Figure 7c). The diagnostic molecules formed by **A3** and **T3** emitted strong fluorescence targeting *Agrobacterium tumefaciens* (Figure 7d). The *Agrobacterium tumefaciens* with only **T3** and **T4** in the two groups did not emit fluorescence, so the influence of the fluorescent probe alone on the production of *Agrobacterium tumefaciens* was excluded (Figure 7e,f). It can be preliminarily judged that among the three diagnostic molecules, the diagnostic molecule formed by **A3** and **T3** has a better detection effect.

### 2.7. Docking of **A1**–**A3** to VirB8

**Figure 8:**
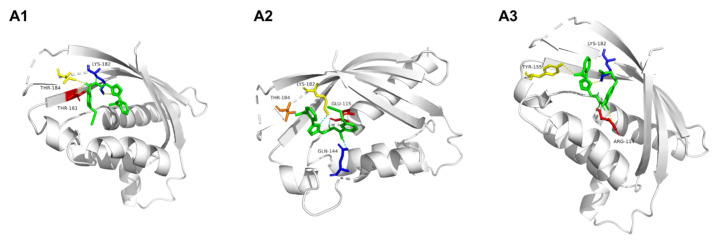
Docked view of compounds **A1**, **A2,** and **A3** at the active site of the enzyme PDB: 4AKY.

The compounds **A1**–**A3** were docked with the receptor VirB8 by AutoDock Vina software, and the docking pocket selected the binding pocket according to the crystal structure of 4AKY (Figure 8 and Table 2). The docking results showed that the binding energy of VirB8 to **A1** was −6.4 kcal/mol, and **A1** interacts with THR 161, LYS 182, and THR184 of VirB8 through hydrogen bonding. The binding energy of VirB8 to **A2** is −7.3 kcal/mol. **A2** interacts with GLU 115, GLN 144, LYS 182, and THR 184 of VirB8 through hydrogen bonding. The binding energy of VirB8 to **A3** is −7.1 kcal/mol, which is hydrogen-bonded to ARG 114, TYR 155, and LYS 182 of VirB8. After docking, all compounds formed three to four hydrogen bonds with amino acid residues in the docking pocket of the VirB8 target protein, among which LYS 182 was the key amino acid residue, which could form hydrogen bond interactions with all compounds. The interaction between **A2** and VirB8 was much stronger than that between **A1** and **A3**.

## 3. Materials and Methods

### 3.1. Apparatus and Characterization

The ^1^H NMR and ^13^C NMR spectra were recorded at 25 °C in a Nuclear Magnetic Resonance Spectrophotometer (DRX-400, Bruker, Bremerhaven, Germany) at 400 MHz for ^1^H NMR in DMSO-*d_6_* and 100 MHz for ^13^C NMR in CDCl_3_. Thin layer chromatography (TLC) was carried out on silica gel (GF254, Qingdao Haiyang Chemical Co., Ltd., Qingdao, China), and the spots were visualized with UV light at 254 nm. Fluorescence spectra were recorded on a fluorescence spectrophotometer (F-2710, Hitachi, Tokyo, Japan). UV-Vis spectrophotometer (UH5300, Hitachi, Tokyo, Japan) was used for the absorption measurements and the spectra were typically measured in the range of 190–1100 nm.

### 3.2. Synthesis

#### 3.2.1. General Procedure for Synthesis of the Compounds (**A1**–**A3**)

##### (E)-2-hydroxy-N’-((5-(2-nitrophenyl)furan-2-yl)methylene)-5-(prop-2-ynyloxy)benzohydrazide (**A1**)

O-Nitroaniline (690.65 mg, 5 mmol), 15% hydrochloric acid solution (3 mL), and distilled water (4.5 mL) were heated in an oil bath at 100 °C for 1 h. After cooling to room temperature, 30% NaNO_2_ (1.2 mL) was added dropwise in an ice bath. Next, the mixture of furfural (480.4 µL, 5 mmol) and copper chloride (134 mg, 1 mmol) was added, and it reacted overnight. After filtering, the residual solid species were removed by extraction in anhydrous ethanol and a crude residue **B** (582 mg, 84%) was acquired.

Methyl 2,5-dihydroxybenzoate (3.36 g, 20 mmol), anhydrous acetone (50 mL), K_2_CO_3_ (6.63 mg, 48 mmol), and 3-bromopropyne (0.86 mL, 10 mmol) were stirred and heated under reflux for 12 h (monitored by TLC). The residual K_2_CO_3_ was removed by filtering, and the solvent was removed by vacuum spin evaporation; the organic mixture was purified by column chromatography over silica gel (ethyl acetate/petroleum ether = 1/3), and product **1** (2.31 g, 85%) was acquired.

Product **1** (2.3 mg, 11.2 mmol), hydrazine hydrate (1 mL, 20 mmol), and anhydrous methanol (20 mL) were stirred and heated under reflux overnight (monitored by TLC). After cooling down to room temperature, distilled water (20 mL) was added. The reaction mixture was extracted with CH_2_Cl_2_ (3 × 20 mL). The combined organic layers were dried over MgSO_4_, and a crude residue product **2** (1.8 g, 92%) was acquired.

Product **2** (1.5 g, 7 mmol), product **B** (1.519 g, 7 mmol), and anhydrous methanol (10 mL) were stirred under reflux for 2 h. After filtering, the residual solid species were removed by extraction in anhydrous ethanol and acquired compound **A1** (1.1 g, 92%).

Yellow solid; m.p. 161–163 °C; ^1^H NMR (400 MHz, DMSO-*d_6_*): δ 11.80 (s, 1H, NH), 11.31 (s, 1H, OH), 8.38 (s, 1H, CH), 7.97 (d, *J* = 8.00 Hz, 1H, CH), 7.91 (d, *J* = 7.60 Hz, 1H, CH), 7.80 (t, *J* = 7.60 Hz, 1H, ArH), 7.65 (t, *J* = 7.60 Hz, 1H, ArH), 7.47 (s, 1H, ArH), 7.13–7.16 (m, 1H, ArH), 7.05 (s, 1H, ArH), 6.94 (d, *J* = 9.20 Hz, 1H, CH), 4.78 (s, 2H, CH_2_), 3.58 (s, 1H, CH); ^13^C NMR (100 MHz, DMSO-*d_6_*) δ: 161.11, 148.09, 138.71, 133.74, 130.03, 124.66, 123.01, 118.62, 116.97, 112.96, 70.03, 22.18, 11.16, 1.03; MS calculated value: 405.10; MS-ESI (*m*/*z*): 404.30 {[M − H]^−^}; Anal. calcd for C_21_H_15_N_3_O_6_: C 62.22, H 3.73, N 10.37, O 23.68; found C 62.28, H 3.92, N 10.93, O 23.91.

##### (E)-N’-((5-(2-nitrophenyl)furan-2-yl)methylene)-3-(prop-2-ynylamino)benzohydrazide (**A2**)

Ethyl 3-aminobenzoate (3.3 g, 20 mmol), K_2_CO_3_ (6.63 mg, 48 mmol), 3-bromopropyne (1.72 mL, 20 mmol), and anhydrous acetone (30 mL) were heated for 12 h (monitored by TLC). The residual K_2_CO_3_ was removed by filtering, and the solvent was removed by vacuum spin evaporation; the organic mixture was purified by column chromatography over silica gel (ethyl acetate/petroleum ether = 1/3), and product **3** (2.31 g, 75%) was acquired.

Product **3** (2.3 g, 11.3 mmol), hydrazine hydrate (1 mL, 20 mmol), and anhydrous methanol (30 mL) were stirred and heated under reflux overnight (monitored by TLC). After cooling down to room temperature, distilled water (30 mL) was added. The reaction mixture was extracted with CH_2_Cl_2_ (3 × 20 mL). The combined organic layers were dried over Na_2_SO_4_, and a crude residue product **4** (1.5 g, 68%) was acquired.

Product **4** (1.5 g, 7.8 mmol), product **B** (1.519 g, 7 mmol), and anhydrous methanol (10 mL) were stirred under reflux for 2 h. After filtering, the residual solid species were removed by extraction in anhydrous ethanol, and compound **A2** (1.1 g, 92%) was acquired.

Yellow solid; m.p. 190–191 °C; ^1^H NMR (400 MHz, DMSO-*d_6_*): δ 11.74 (s, 1H, NH), 8.38 (s, 1H, ArH), 7.89–7.97 (m, 2H, ArH), 7.79 (t, *J* = 7.2 Hz, 1H,ArH), 7.65 (t, *J* = 7.2 Hz, 1H, ArH), 7.65 (t, *J* = 7.2 Hz, 1H, ArH), 7.26 (t, *J* = 7.2 Hz, 1H, ArH), 7.05–7.14 (m, 4H, ArH, CH), 6.86 (s, 1H, ArH), 6.72 (s, 1H, NH), 6.85 (d, *J* = 7.2 Hz, 1H, CH), 6.30 (s, 1H, NH), 3.94 (s, 2H, CH_2_), 3.09 (s, 1H, CH); ^13^C NMR (100 MHz, DMSO-*d*_6_) δ:151.29, 149.86, 148.34, 147.45, 137.38, 136.28, 133.23, 129.79, 129.31, 124.72, 122.87, 116.40, 115.95, 114.99, 112.72, 82.37, 73.61, 32.40; MS calculated value: 388.12; MS-ESI (*m*/*z*): 387.21 {[M − H]^−^}; Anal. calcd for C_21_H_16_N_4_O_4_: C 64.94, H 4.15, N 14.43, O 16.48; found C 65.13, H 4.22, N 14.53, O 16.71.

##### (E)-5-(2-azidoethoxy)-2-hydroxy-N’-((5-(2-nitrophenyl)furan-2-yl)methylene)benzohydrazide (**A3**)

Methyl 2,5-dihydroxybenzoate (3.36 g, 20 mmol), 1-Bromo-2-chloroethane (844 µL, 10 mmol), K_2_CO_3_ (2.76 g, 20 mmol), and anhydrous acetone (30 mL) were stirred under reflux for 14 h (monitored by TLC). The residual K_2_CO_3_ was removed by filtering, and the solvent was removed by vacuum spin evaporation; the organic mixture was purified by column chromatography over silica gel (ethyl acetate/petroleum ether = 1/5), and product **5** (2.87 g, 84%) was acquired.

Product **5** (2.5 g, 11 mmol), hydrazine hydrate (500 µL, 11 mmol), and anhydrous methanol (30 mL) were stirred under reflux overnight. After cooling down to room temperature, distilled water (20 mL) was added, and the reaction mixture was extracted with CH_2_Cl_2_ (3 × 20 mL). The combined organic layers were dried over Na_2_SO_4_, and a crude residue product **6** (1.8 g, 78%) was acquired.

Product **6** (1.5 g, 6.5 mmol), product **B** (1.5 g, 7 mmol), and anhydrous methanol (10 mL) were stirred under reflux for 2 h and filtered, and product **7** (1.2 g, 92%) was acquired.

Product **7** (1 g, 2.32 mmol), sodium azide (325 mg, 5 mmol), and N,N-dimethylformamide (15 mL) were stirred at 60 °C overnight; distilled water (20 mL) was added. The reaction mixture was extracted with CH_2_Cl_2_ (3 × 20 mL). The combined organic layers were dried over Na_2_SO_4_. The solvent was removed by vacuum spin evaporation. The organic mixture was purified by column chromatography over silica gel (ethyl acetate/petroleum ether = 1/5), and compound **A3** (800 mg, 80%) wasd acquired.

Brown solid; m.p. 202–204 °C; ^1^H NMR (400 MHz, DMSO-*d_6_*): δ 11.82 (s, 1H, CH), 11.35 (s, 1H, NH), 8.39 (s, 1H ArH), 7.98 (d, *J* = 5.2 Hz, 1H, ArH), 7.91 (d, *J* = 5.2 Hz, 1H, CH), 7.80 (t, *J* = 5.2 Hz, 1H, ArH), 7.65 (t, *J* = 5.2 Hz, 1H, ArH), 7.45 (s, 1H, ArH), 7.10–7.14 (m, 2H, ArH), 7.06 (s, 1H ArH), 6.95 (d, *J* = 6.0 Hz, 1H, CH), 4.16 (m, 2H, CH_2_), 3.66 (m, 2H, CH_2_); ^13^C NMR (100 MHz, DMSO-*d_6_*) δ: 164.62, 153.63, 150.34, 147.52, 138.54, 133.27, 130.41, 129.86, 124.70, 121.72, 118.69, 115.86, 113.83, 112.80, 67.96, 50.13; MS calculated value: 436.11; MS-ESI (*m*/*z*): 435.33 {[M − H]^−^}; Anal. calcd for C_20_H_16_N_6_O_6_: C 55.05, H 3.70, N 19.26, O 22.00; found C 55.23, H 3.92, N 19.93, O 23.31.

#### 3.2.2. General Procedure for the Synthesis of the Probes (**T1**–**T4**)

##### (E)-2-((2-(2-nitrobenzyloxy)phenylimino)methyl)-5-(2-azidoethoxy)phenol (**T1**)

2,4-Dihydroxybenzaldehyde (828.72 mg, 6 mmol), 1,3-Dibromopropane (1.6 g, 8 mmol), NaHCO_3_ (504 mg, 6 mmol), and anhydrous acetone (30 mL) were stirred at 60 °C for 48 h. After cooling down to room temperature, the residual NaHCO_3_ was removed by filtering, and the solvent was removed by vacuum spin evaporation; the organic mixture was purified by column chromatography over silica gel (ethyl acetate/petroleum ether = 1/5), and product **8** (723 mg, 84%) was acquired.

In the presence of formic acid (2 d), product **8** (700 mg, 2.7 mmol), 2-Aminophenol (294.3 mg, 2.7 mmol), and anhydrous ethanol (15 mL) were stirred under reflux at 90 °C for 2 h and filtered, and product **9** (650 mg, 86%) was acquired.

Product **9** (600 mg, 1.7 mmol), K_2_CO_3_ (264 mg, 1.7 mmol), 2-Nitrobenzyl Bromide (363.8 mg, 1.7 mmol), and anhydrous acetone (15 mL) were stirred under reflux with N_2_ for 12 h, cooled down to room temperature, and evaporated under vacuum; the mixture of sodium hydroxide (0.2 mmol) and water (100 mL) was added. The reaction mixture was extracted with ethyl acetate. The combined organic layers were washed with saturated brine until neutral and dried over Na_2_SO_4_. Filtered and evaporated, the organic mixture was purified by column chromatography over silica gel (ethyl acetate/petroleum ether = 1/5), and product **10** (459 mg, 73%) was acquired.

Product **10** (400 mg, 0.94 mmol), sodium azide (292.5 mg, 4.5 mmol), and N, N-dimethylformamide (10 mL) were stirred at 60 °C for 8 h. After cooling down to room temperature, distilled water (20 mL) was added; the reaction mixture was extracted with CH_2_Cl_2_ (3 × 20 mL). The combined organic layers were dried over Na_2_SO_4_, and probe **T1** (380 mg, 84%) was acquired.

Brown red solid; m.p. 160–161 °C; ^1^H NMR (400 MHz, DMSO-*d_6_*): δ 9.00 (s, 1H, OH), 7.67 (d, *J* = 8.0 Hz, 1H, ArH), 7.59 (t, *J* = 8.0 Hz, 1H, ArH), 7.52 (d, *J* = 8.0 Hz, 1H, ArH), 7.43 (d, *J* = 8.0 Hz, 1H, ArH), 6.97–7.02 (m, 1H, ArH), 6.92–6.96 (m, 1H, ArH), 6.85–6.89 (m, 1H, ArH), 6.44 (s, 1H, CH), 6.20(s, J = 2.4 Hz, 1H, ArH), 6.08–6.14 (m, 1H, ArH), 4.49 (t, *J* = 6.0 Hz, 2H, CH_2_), 4.01 (d, *J* = 6.0 Hz, 2H, CH_2_), 3.51 (t, *J* = 8.0 Hz, 2H, CH_2_), 2.01–2.07 (m, 2H, CH_2_); ^13^C NMR (100 MHz, DMSO-*d_6_*) δ: 154.01 (t, *J* = 6.0 Hz, 2H, CH_2_), 9.18, 156.34, 149.66, 142.96, 135.17, 132.79, 130.27, 129.84, 129.72, 123.97, 122.01, 117.31, 117.17, 116.35, 115.16, 105.71, 101.87, 74.78, 64.86, 48.16, 40.26, 40.12, 39.38, 39.84, 39.70, 39.56, 28.54; MS calculated value: 437.14; MS-ESI (*m*/*z*): 438.0146 {[M + H]^+^}; Anal. calcd for C_23_H_21_N_5_O_5_: C 61.74, H 4.73, N 15.65, O 17.88; found C 62.23, H 4.92, N 15.93, O 18.01.

##### (E)-2-((2-hydroxyphenylimino)methyl)-5-(prop-2-ynyloxy)phenol (**T2**)

2,4-Dihydroxybenzaldehyde (414 mg, 3 mmol), 3-bromopropyne (238 µL, 3 mmol), Na_2_CO_3_ (504 mg, 6 mmol), and anhydrous acetone (10 mL) were stirred under reflux at 60 °C for 12 h. After cooling down to room temperature, the residual Na_2_CO_3_ was removed by filtering and the solvent was removed by vacuum spin evaporation. The organic mixture was purified by column chromatography over silica gel (ethyl acetate/petroleum ether = 1/5), and product **11** (323 mg, 83%) was acquired.

In the presence of formic acid (2 d), product **11** (300 mg, 1.7 mmol), 2-Aminophenol (294.3 mg, 2.7 mmol), and anhydrous ethanol (15 mL) were stirred under reflux at 90 °C for 2 h and filtered, and probe **T2** (254 mg, 96%) was acquired.

Golden yellow solid; m.p. 242–244 °C; ^1^H NMR (400 MHz, CDCl_3_): δ 14.42 (s, 1H, OH), 9.75 (s, 1H, OH), 8.87 (s, 1H, CH), 7.49 (d, 1H, *J* = 8.8 Hz, ArH), 7.35 (d, 1H, *J* = 8.0 Hz, ArH), 7.10 (t, 1H, *J* = 7.2 Hz, ArH), 6.95 (d, 1H, *J* = 7.6 Hz, ArH), 6.87 (t, 1H, *J* = 7.6 Hz, ArH), 6.41–6.52 (m, 2H, ArH), 4.85 (s, 1H, CH_2_), 3.62 (s, 1H, CH); ^13^C NMR (100 MHz, DMSO-*d_6_*) δ: 165.62, 162.01, 160.47, 150.98, 134.43, 134.30, 127.92, 120.09, 119.54, 116.86, 113.97, 107.39, 102.54, 79.31, 79.05, 56.08; MS calculated value: 267.09; MS-ESI (*m*/*z*): 268.0332 {[M + H]^+^}; Anal. calcd for C_16_H_13_NO_3_: C 71.90, H 4.90, N 5.24, O 17.96; found C 72.03, H 4.98, N 5.93, O 18.21.

##### 2-(benzo[d]thiazol-2-yl)-5-(prop-2-ynyloxy)phenol (**T3**)

Product **11** (700 mg, 4 mmol), 2-Aminophenol (500 mg, 4 mmol), DMSO (10 mL), and AgNO_3_ (18 mg) were stirred. A change in color of the solution to reddish brown after the inferred irradiation was observed. Filtered and put under infrared light to dry overnight, the organic mixture was purified by column chromatography over silica gel (ethyl acetate/petroleum ether = 1/5), and probe **T3** (603 mg, 82%) was acquired.

Yellow solid; m.p. 179–180 °C; ^1^H NMR (400 MHz, CDCl_3_): δ 7.88 (d, *J* = 8.00 Hz, 1H, ArH), 7.81 (d, *J* = 7.60 Hz, 1H, ArH), 7.54 (d, *J* = 8.80 Hz, 1H, ArH), 7.42 (t, *J* = 8.00 Hz, 1H, ArH), 7.31 (t, *J* = 7.20 Hz, 1H, ArH), 6.61 (s, 1H, ArH), 6.54 (d, *J* = 8.80 Hz, 1H, ArH), 4.68 (s, 2H, CH_2_), 2.50 (s, 1H, CH); ^13^C NMR (100 MHz, DMSO-*d_6_*) δ: 166.00, 161.07, 158.44, 151.84, 133.98, 130.27, 125.26, 122.42, 122.14, 112.20, 108.18, 102.29, 79.30, 79.10, 55.75; MS calculated value: 281.05; MS-ESI (*m*/*z*): 280.0267 {[M − H]^−^}; Anal. calcd for C_16_H_11_NO_2_S: C 68.31, H 3.94, N 4.98, O 11.37, S 11.40; found C 68.53, H 4.12, N 5.03, O 11.41, S 11.61.

##### (E)-5-(3-azidopropoxy)-2-((2-hydroxyphenylimino)methyl)phenol (**T4**)

Product **8** (400 mg, 1.55 mmol), sodium azide (292.5 mg, 4.5 mmol), and N, N-dimethylformamide (10 mL) were stirred at 60 °C for 8 h. After cooling down to room temperature, distilled water (20 mL) was added; the reaction mixture was extracted with CH_2_Cl_2_ (3 × 20 mL). The combined organic layers were dried over Na_2_SO_4_, and product **12** (350 mg, 84%) was acquired.

In the presence of formic acid (2 d), product **12** (300 mg, 1.45 mmol), 2-Aminophenol (158 mg, 1.45 mmol), and anhydrous ethanol (10 mL) were stirred under reflux at 90 °C for 2 h, filtered, and put under infrared light to dry overnight, and probe **T4** (230 mg, 86%) was acquired.

Yellow solid; m.p. 181–183 °C; ^1^H NMR (400 MHz, CDCl_3_): δ 8.57 (s, 1H, CH), 7.32 (d, *J* = 8.40 Hz, 1H, ArH), 7.19 (t, *J* = 7.60 Hz, 1H, ArH), 7.14 (d, *J* = 6.80 Hz, 1H, ArH), 7.05 (d, *J* = 8.40 Hz, 1H, ArH), 6.95 (t, *J* = 7.60 Hz, 2H, ArH), 6.51–6.55 (m, 2H, ArH), 4.11 (t, *J* = 6.00 Hz, 2H, CH_2_), 3.53 (t, *J* = 6.40 Hz, 2H, CH_2_), 2.08 (m, 2H, CH_2_); ^13^C NMR (100 MHz, DMSO-*d_6_*) δ: 165.83, 163.22, 160.41, 150.95, 134.43, 134.24, 127.77, 119.91, 119.40, 116.82, 113.56, 107.22, 102.00, 65.26, 48.11, 28.47; MS calculated value: 312.12; MS-ESI (*m*/*z*): 311.22 {[M − H]^−^}; Anal. calcd for C_16_H_16_N_4_O_3_: C 61.53, H 5.16, N 17.94, O 15.37; found C 61.93, H 5.92, N 13.23, O 15.71.

### 3.3. Determination of the UV Absorption Wavelength of Inhibitors

The sample of the compounds (**A1**–**A3**) was diluted to 1 × 10^−5^ mg/mL. After thorough mixing, the target molecules were detected using a UV spectrophotometer at UV absorption wavelengths. The volume of the sample needed to be greater than 2/3 of the cuvette volume. All experiments were performed in triplicate.

### 3.4. Fluorescence Intensity Measurement by Fluorescence Spectrophotometer

The sample of probes (**T1**–**T4**) was diluted to 1 × 10^−5^ mg/mL for fluorescence, excitation, and emission spectra measurement by a fluorescence spectrophotometer. All experiments were performed in triplicate.

### 3.5. In Vitro Validation of the “Click” Reaction and Optimization of Conditions

In this study, the “click” reaction was successfully completed in the organism by simulating the environment. The ratio of reactant concentration, reaction time, and catalyst dosage was investigated in 0.5% PBS solution at 37 °C.

**Figure 9:**
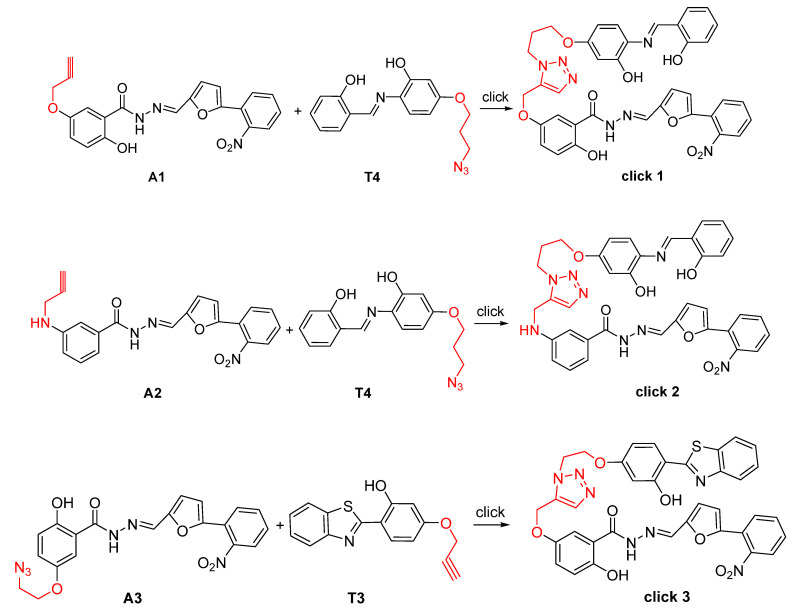
The chemical synthesis routes of the click reactions.

First, 10 µL **T4** solutions with different molar concentrations were prepared, i.e., 30, 60, and 90 µmol/L and mixed with 10 μL **A1** (30 µmol/L) in the 0.5 mL of 0.5% PBS, respectively. Then, 15 µL of sodium ascorbate (10 µmol/L) and 15 µL of copper sulfate solution (10 µmol/L) were successively added. After mixing, the reaction was carried out at 37 °C for 30 min and 60 min, and click 1 reaction formation was detected by HPLC-MS, respectively. The responses of click 2 and click 3 were the same as above, and three replicates were set in parallel for each group. HPLC was used to test the peak area under different ratios of reactant concentration, reaction time, and catalyst dosage (Figure 9).

### 3.6. Cytotoxicity: MTT Cell Proliferation Assay

The “click” reaction conditions in click chemistry require copper sulfate and sodium ascorbate as catalysts, and the copper sulfate concentration is generally too high, which causes certain damage to the body. RAW264.7 cells were used as test cells to explore the safe concentration of copper sulfate, compounds **A1**–**A3,** and probes **T1**–**T4**. The macrophage suspension of RAW264.7 cells in the logarithmic growth stage was adjusted to 5 × 10^4^ cells/mL and seeded in 96-well plates with 100 µL in each well. The cells were incubated at 37 °C and 5% CO_2_ for 24 h and then treated with different concentrations of copper sulfate solution (each concentration was repeated three times). After being treated with copper sulfate for 36 h, 10 µL MTT (5 mg/mL) was added to each well, and the cells were incubated at 37 °C and 5% CO_2_ for 4 h. Then, the MTT in the well was discarded, and 100 µL DMSO was added to each well and incubated at 37 °C for 0.5 h until the crystals were completely dissolved. The microplate reader read the OD490 nm of each well. The cytotoxicity assays of compounds **A1**–**A3** and probes **T1**–**T4** were the same as above. The cell proliferation inhibition rate (IC_50_) was calculated using SPSS 16.0 software.

### 3.7. Antibacterial Activity on Agrobacterium tumefaciens

The compounds **A1**–**A3** were evaluated through the plate microdilution test according to the guidelines of the Clinical and Laboratory Standards Institute (CLSI). A colony of *Agrobacterium tumefaciens* grown on lysogeny broth (LB) agar plates was inoculated in lysogeny broth (LB) and incubated in an aerobic environment at 28 °C overnight (ON). The bacterial suspension was diluted to 5 × 10^7^ CFU/mL, and 200 µL was added to each well, obtaining a final density of 2.5 × 10^7^ CFU/well. Meanwhile, **A1**–**A3** were serially diluted in 1X PBS at concentrations of 30, 50, and 100 µmol/L. Cefuroxime was used as an antibiotic control and DMSO as a solvent control. Subsequently, the plates were incubated at 28 °C in aerobiosis, and the growth rate was evaluated after 4, 6, 8, and 10 h using an ultraviolet (UV) detector.

### 3.8. Fluorescent Detection of Agrobacterium tumefaciens by Diagnostic Molecules

An amount of 3 mL of the standby Agrobacterium solution at a concentration of 5 × 10^3^ CFU/mL was added to the shaker tube. Then, 200 µL of **A1** at a concentration of 30 µmol/L, **A2** at a concentration of 50 µmol/L, and **A3** at a concentration of 50 µmol/L were added at 28 °C. After incubation at 200 RPM/min for 8 h, the upper layer of the medium was poured off after centrifugation and washed three times with PBS, and the bacterial solution was resuspended by adding 1 mL of medium. Then, the same amount of probe was added, and 15 µL copper sulfate and sodium ascorbate were added at a concentration of 10 µmol/L. The control group was treated with only the corresponding probe, and the concentration of each probe was the same as that of the corresponding inhibitor. After adding probes to each group, the “click” reaction was complete for 1 h. After centrifugation, the upper layer of the medium was poured off and washed three times with PBS. Then, 1 mL of the medium was added to resuspend the bacterial solution, and 10 µL was added to the slide for fluorescence microscope detection and observation of the fluorescence of Agrobacterium in each group.

### 3.9. Molecular Docking

The crystal structure of VirB8 (PDB ID: 4AKY) came from the PDB database. The compounds **A1**–**A3** as input for the docking study were drawn by ChemDraw 22, and the PDB file of **A1**–**A3** was generated by StoneMIND Collector. It was connected to the interaction between **A1**–**A3** and VirB8 (PDB ID: 4AKY) through AutoDock Vina 1.2.3 and analyzed by PyMOL 4.6.

## 4. Conclusions

In this study, the compounds **A1**–**A3** were designed and synthesized according to B8I-2 as the basic skeleton, and four fluorescent probes **T1**–**T4** were designed and synthesized according to the excited state intramolecular proton transfer mechanism. The structures of the seven compounds synthesized in this study were confirmed by ^1^H NMR, ^13^C NMR, MS characterization, and elemental analysis. The docking results between the **A1**–**A3** and the receptor VirB8 molecule showed that the docking between **A1**–**A3** and VirB8 was successful, which was basically consistent with the binding sites in the literature, and the binding energy between **A2** and VirB8 was the largest.

The compounds **A1**–**A3** synthesized in this paper had antibacterial activity against *Agrobacterium tumefaciens*. The antibacterial activity of compound **A1**–**A3** was better than that of B8I-2. **A1**–**A3** and **T1**–**T4** underwent the “click” reaction in the physiological condition to form diagnostic molecules. This laid a foundation for the detection of animal pathogenic bacteria in the later stage.

## Figures and Tables

**Table 1:** Results of the cytotoxicity test.

Comp	A1	A2	A3	T1	T2	T3	T4
IC_50_/μmol·L^−1^	>98	>96	>100	>100	>92	>100	>87

**Table 2:** The binding free energy.

Comp	A1	A2	A3
4AKY	6.4 kcal/mol	7.3 kcal/mol	7.1 kcal/mol

## Data Availability

Not applicable.

## Supplementary Materials

The following supporting information can be downloaded at: https://www.mdpi.com/article/10.3390/molecules28062758/s1; Figures S1–S7: ^1^H, ^13^C NMR, and MS of all products; Figures S8–S10: in vitro validation of the “click” reaction and optimization of conditions.

## Sample Availability

Samples of the compounds are available from the authors.
