# Supplementary material for: Click Triazole as a Linker for Pretargeting Strategies: Synthesis, Docking Investigations, Fluorescence Diagnosis, and Antibacterial Action Studies
Source: Molecules. 2023 Mar 18;28(6):2758. doi: 10.3390/molecules28062758 (PMC10057994; doi:10.3390/molecules28062758)
Supplement: Supplementary file 1 [file molecules-28-02758-s001.zip › Supplementary Materials.pdf]

Click Triazole as a Linker for Pretargeting Strategies： Synthesis, Docking Investigations, Fluorescence Diagnosis, and Antibacterial Action Studies

Qian Liu [1], Mingxia Zhao [2], Cairong Song [1], Jiankang Sun [1], Jiali Tao [2], Bin Sun [2], and Junbing Jiang [1,2,*]

[1] Department of Veterinary Medicine, Shanxi Agricultural University, Jinzhong 0030801, China
[2] Department of Mining Engineering, Shanxi Institute of Engineering and Technology, Yangquan 045000, China
* Correspondence: sxndjeffrey@126.com (J.J.)

Supporting information ^{1}H, ^{13}C NMR and MS of all products. Figure S1-S7

The HPLC-MS results for the three "click" reactions. Figure S8-S10

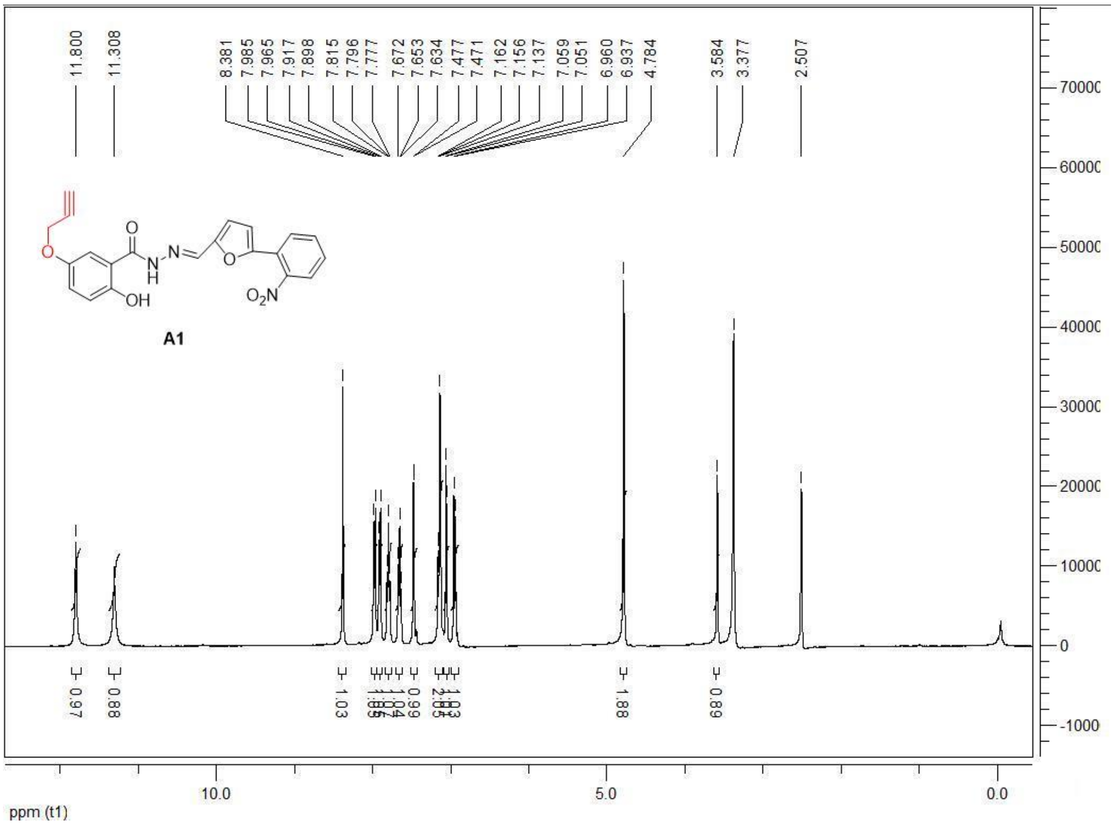
11.800
11.308
8.381
7.985
7.965
7.917
7.898
7.815
7.796
7.777
7.672
7.653
7.634
7.477
7.471
7.162
7.156
7.137
7.059
7.051
6.960
6.937
4.784
3.584
3.377
2.507
A1
0.97
0.88
1.03
0.99
1.88
0.89
10.0
5.0
0.0
ppm (t1)

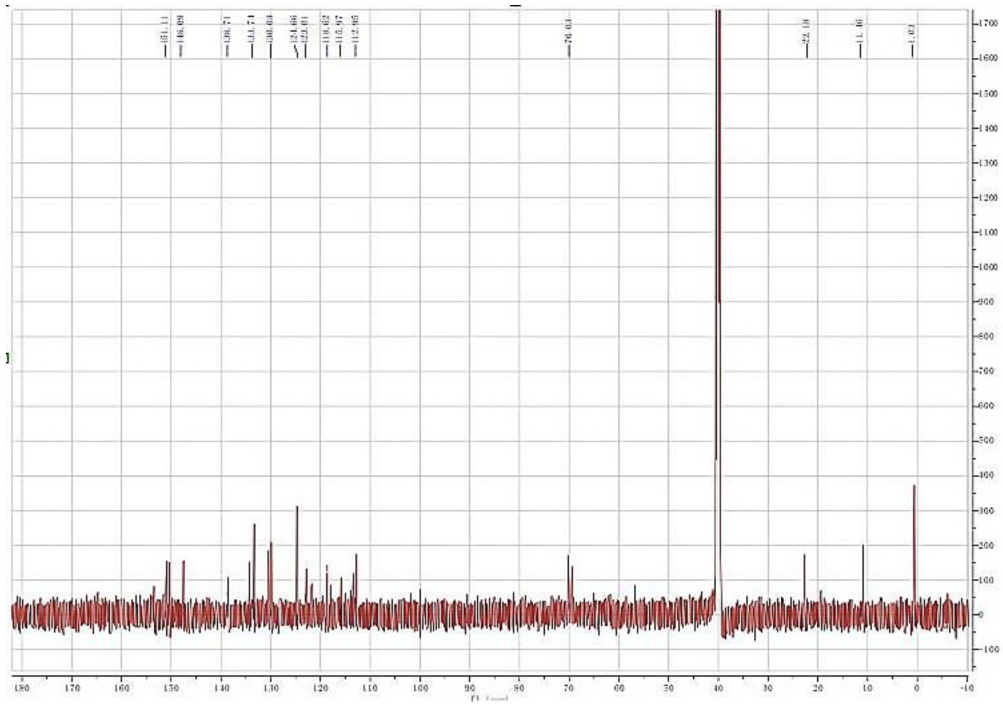

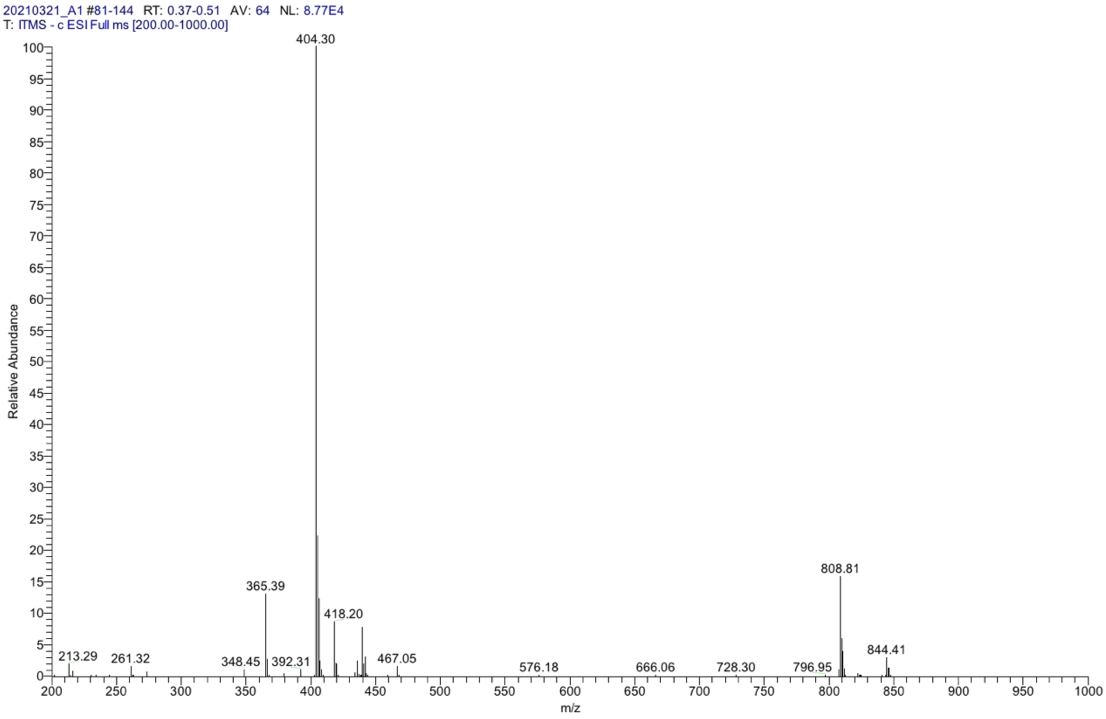

Figure S1. ^{1}H, ^{13}C NMR and MS of **A1**.

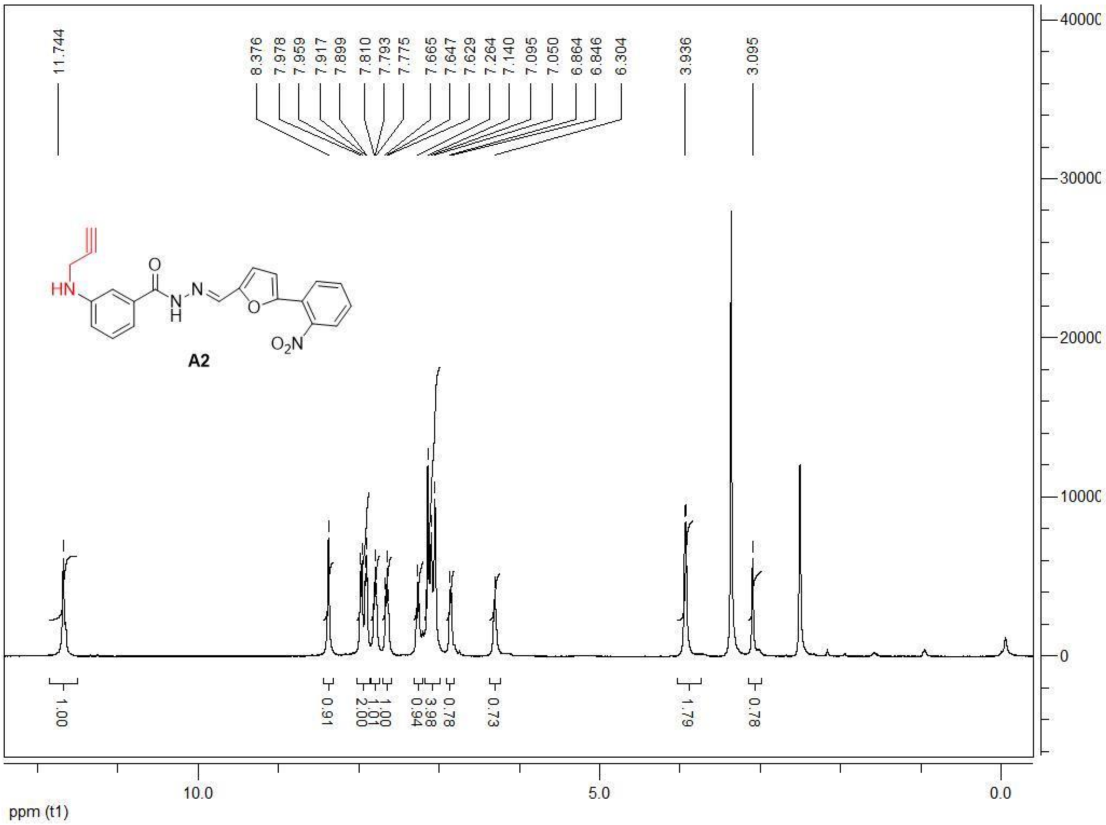

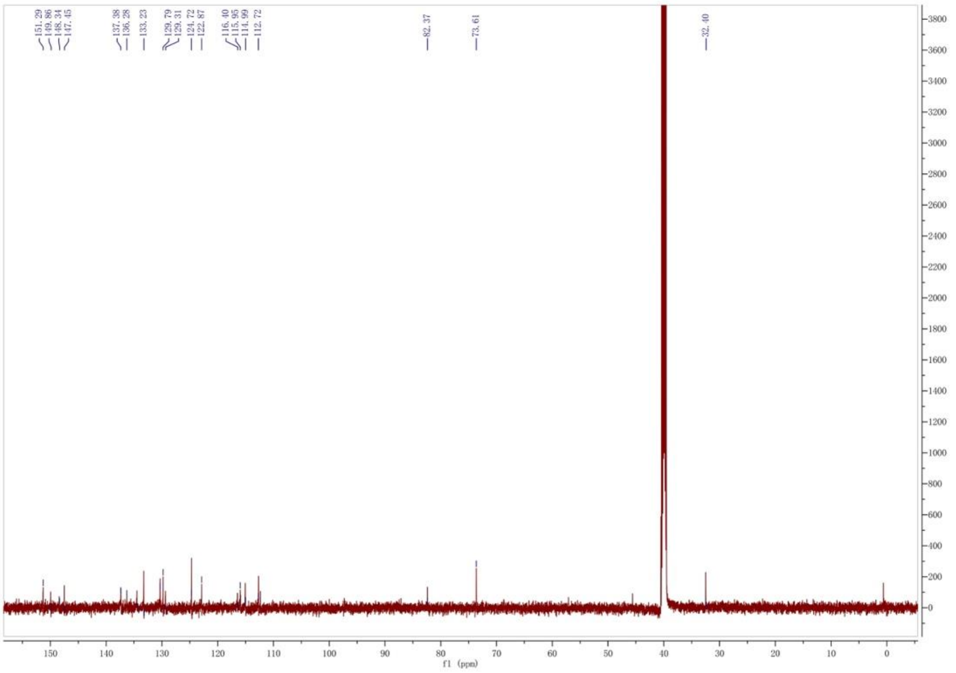

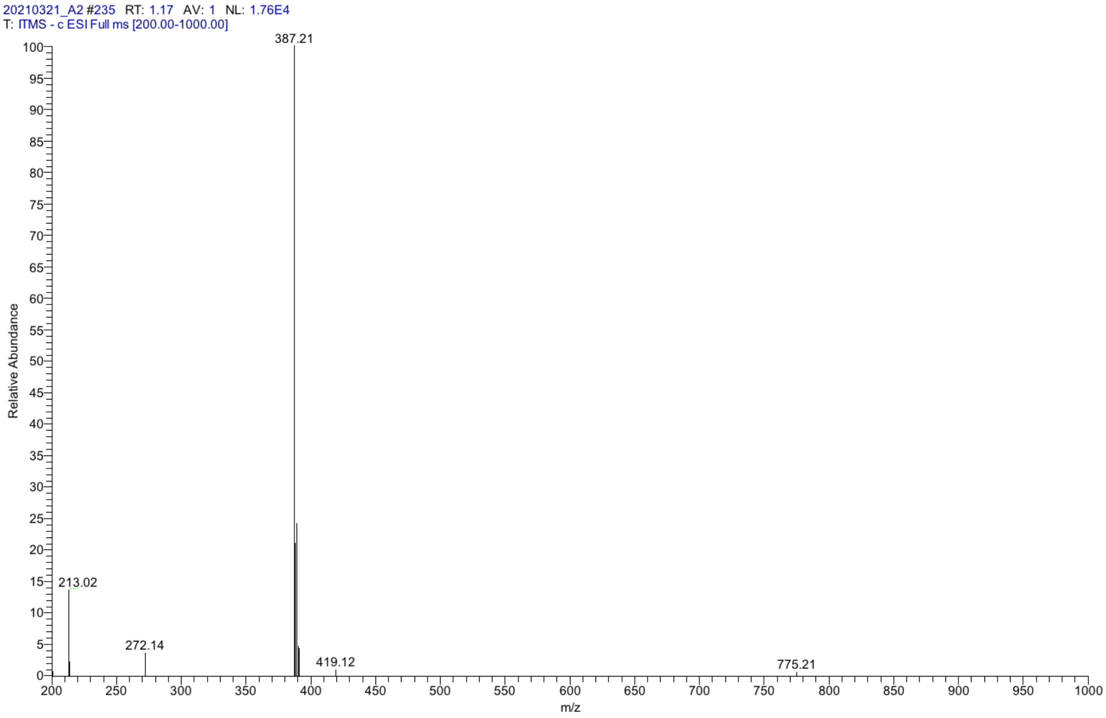

Figure S2. ^{1}H, ^{13}C NMR and MS of **A2**.

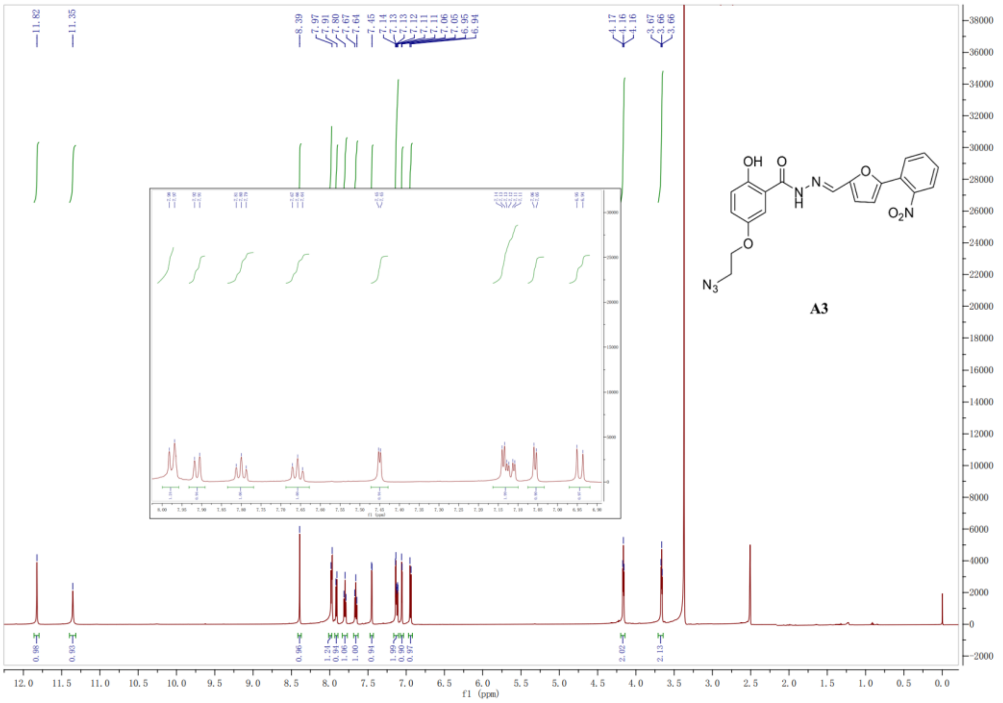

11.82
11.35
8.39
7.97
7.91
7.80
7.67
7.64
7.45
7.14
7.13
7.13
7.12
7.11
7.11
7.06
7.05
6.95
6.94
4.17
4.16
4.16
3.67
3.66
3.66
OH
O
N
H
N
O
O_2N
O
N_3
A3
f1 (ppm)

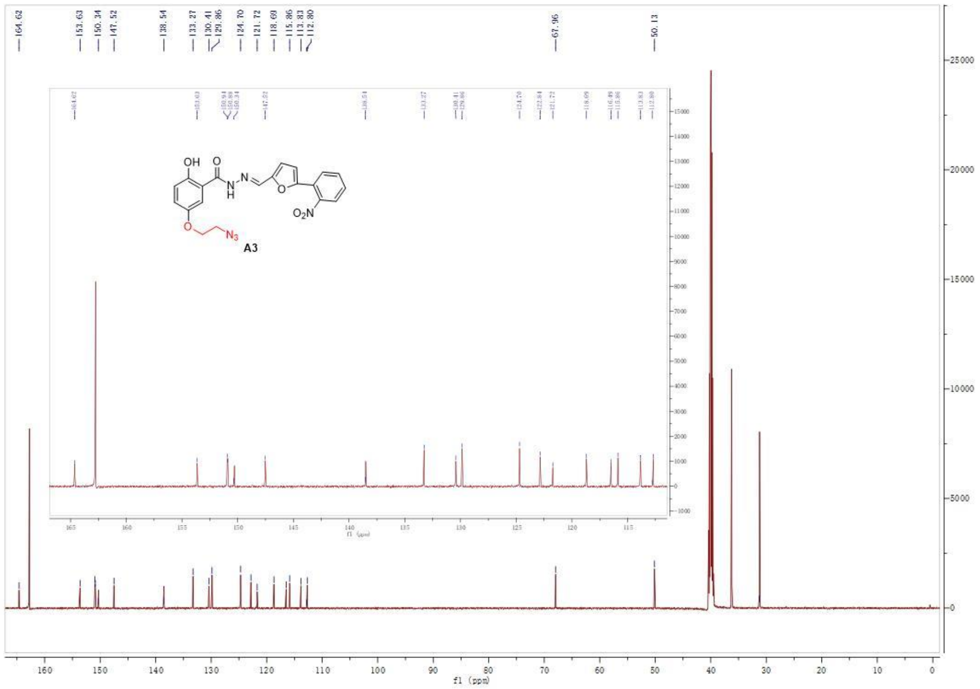

164.62
153.63
150.34
147.52
138.54
133.27
130.41
129.86
124.70
121.72
118.69
115.86
113.83
112.80
67.96
50.13
OH
O
N
H
N
O
O_2N
O
N_3
A3
f1 (ppm)

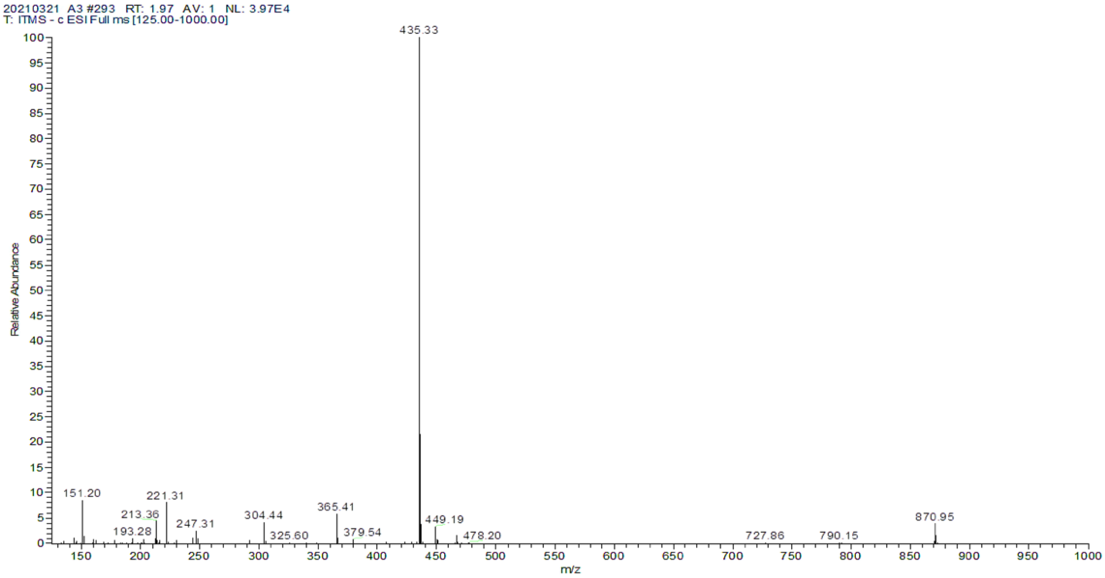

Figure S3. ^{1}H, ^{13}C NMR and MS of **A3**.

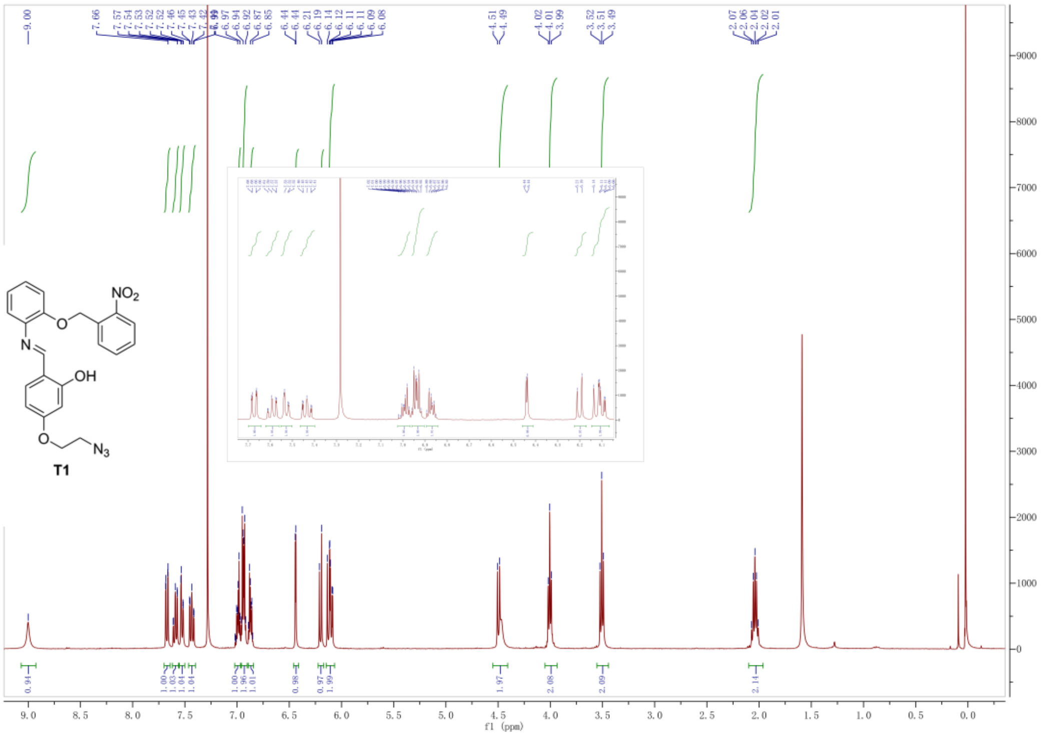

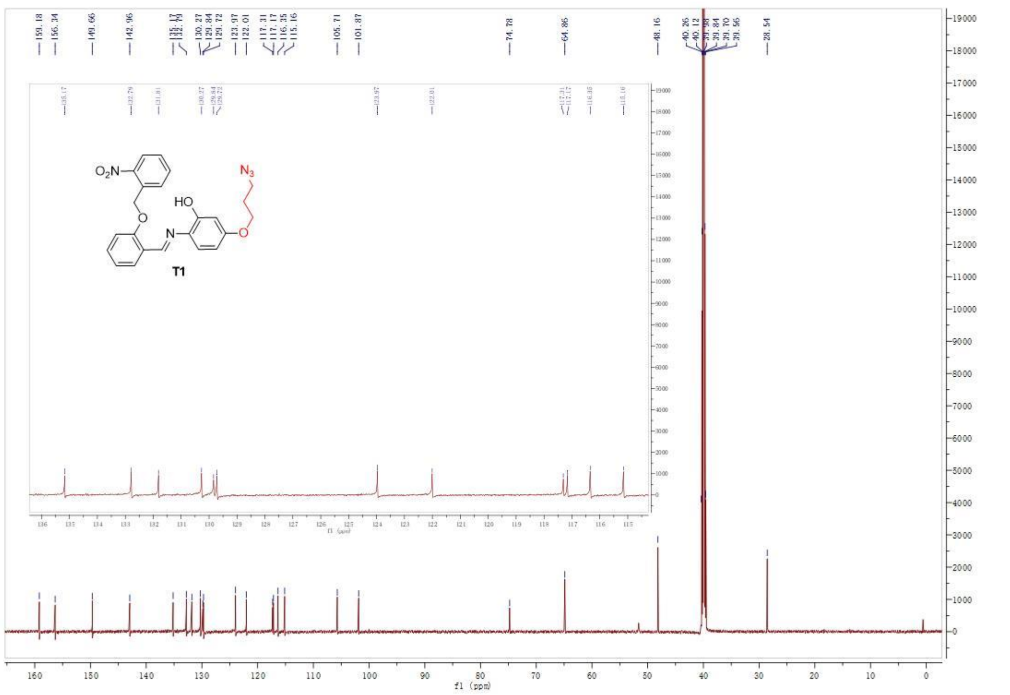

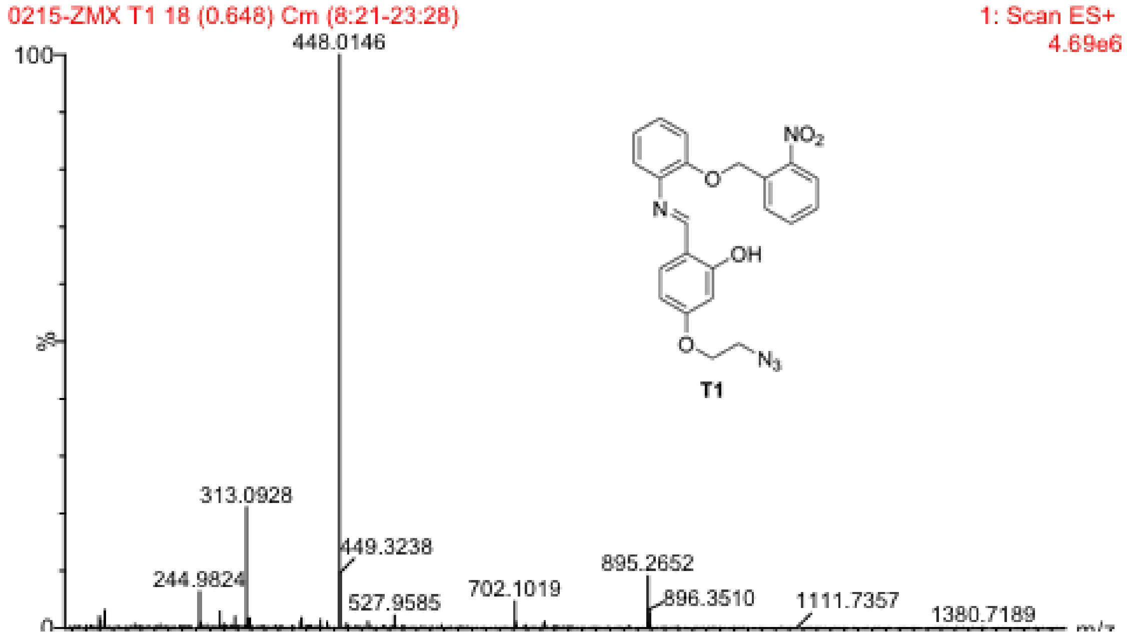

Figure S4. ^{1}H, ^{13}C NMR and MS of **T1**.

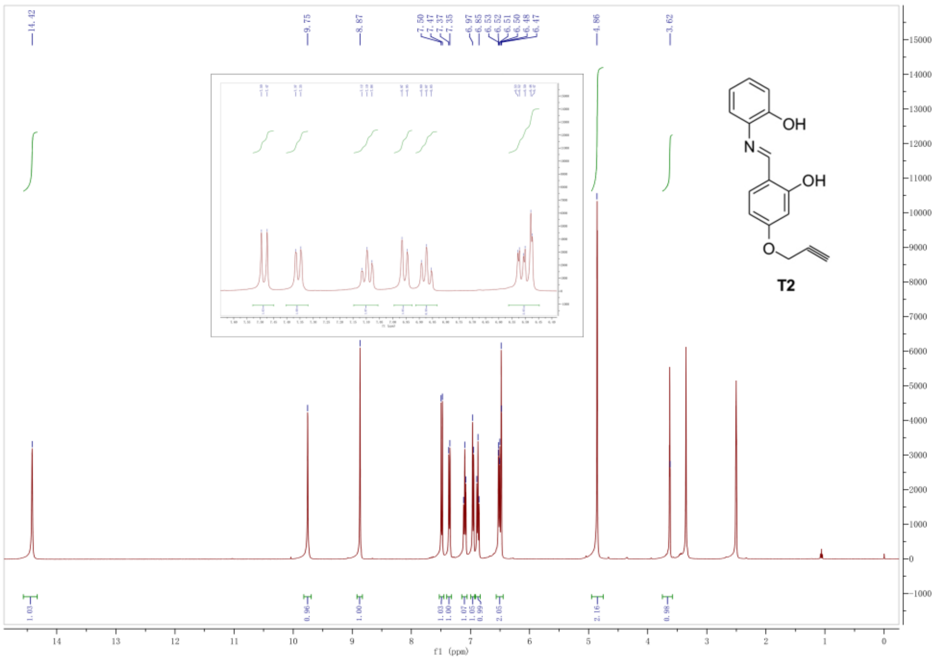
14.42
9.75
8.87
7.50
7.47
7.37
7.35
6.97
6.85
6.53
6.52
6.51
6.50
6.48
6.47
4.86
3.62
OH
N
OH
O
T2
1.03
0.96
1.00
1.03
1.00
1.07
1.05
0.99
2.05
2.16
0.98
f1 (ppm)

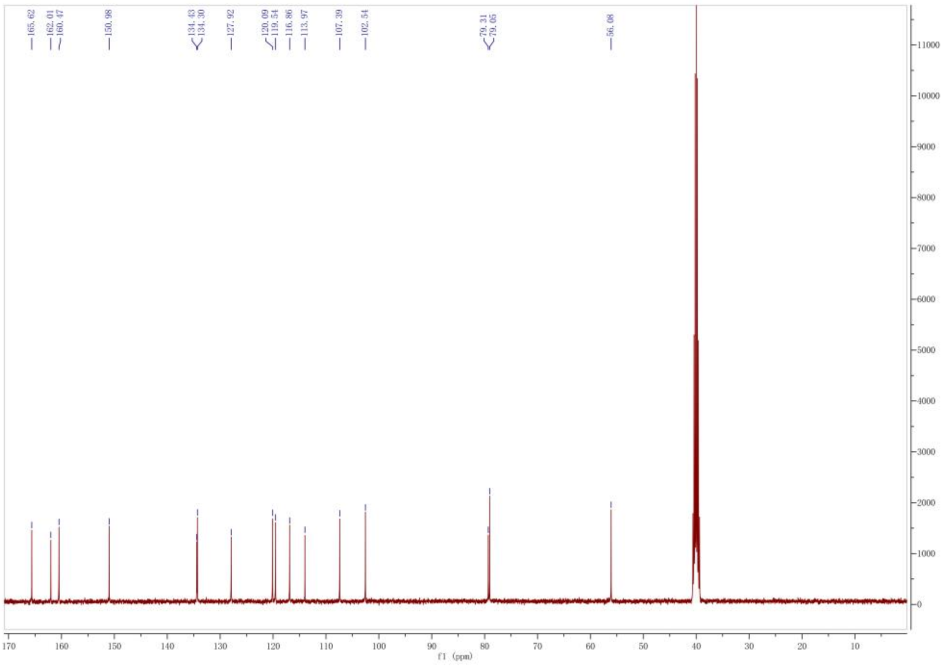
165.62
162.01
160.47
150.98
134.43
134.30
127.92
120.09
119.54
116.86
113.97
107.39
102.54
79.31
79.05
56.08
f1 (ppm)

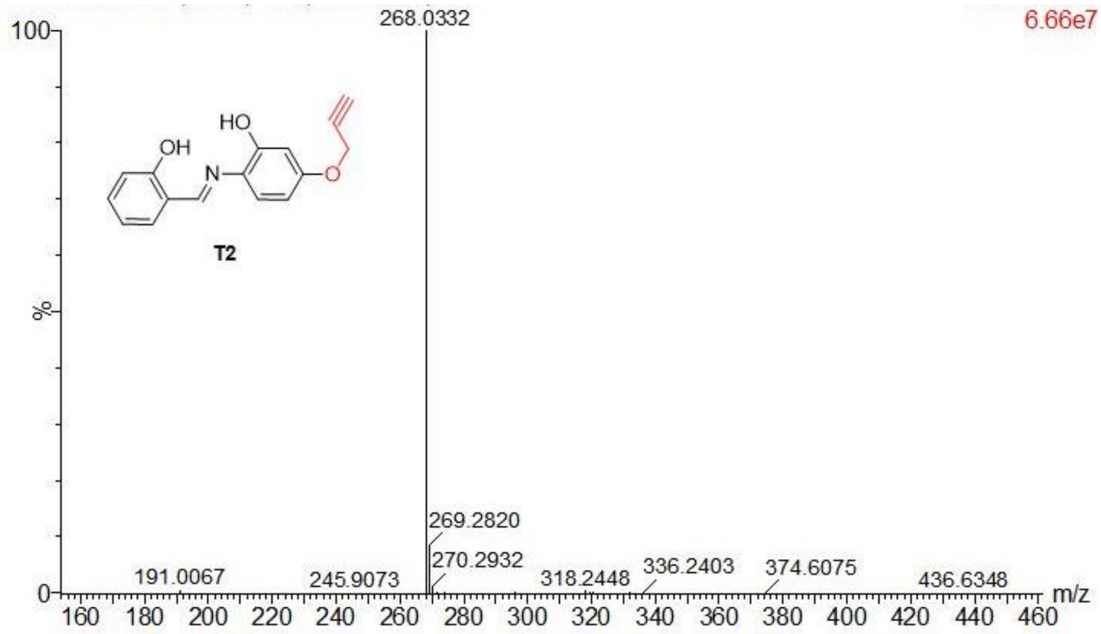

Figure S5. ^{1}H, ^{13}C NMR and MS of **T2**.

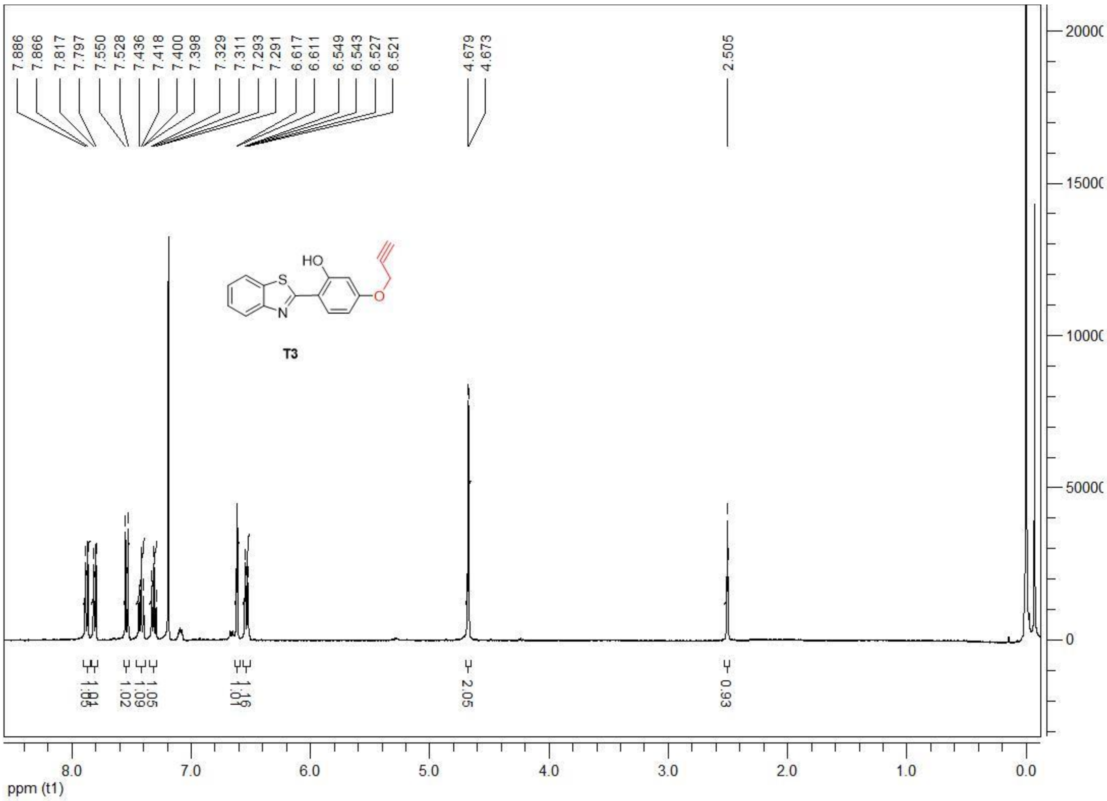

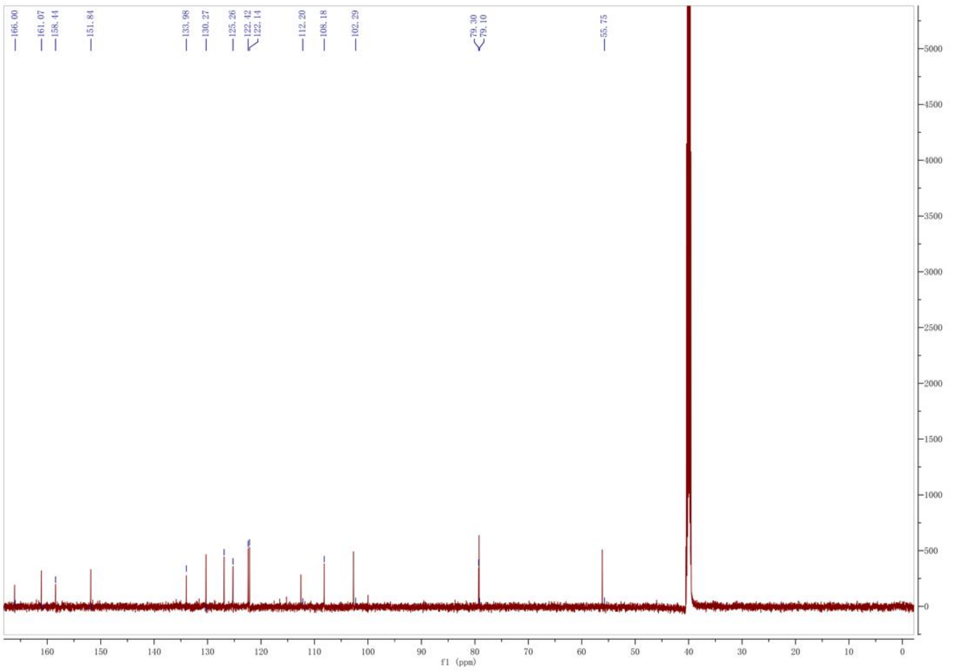

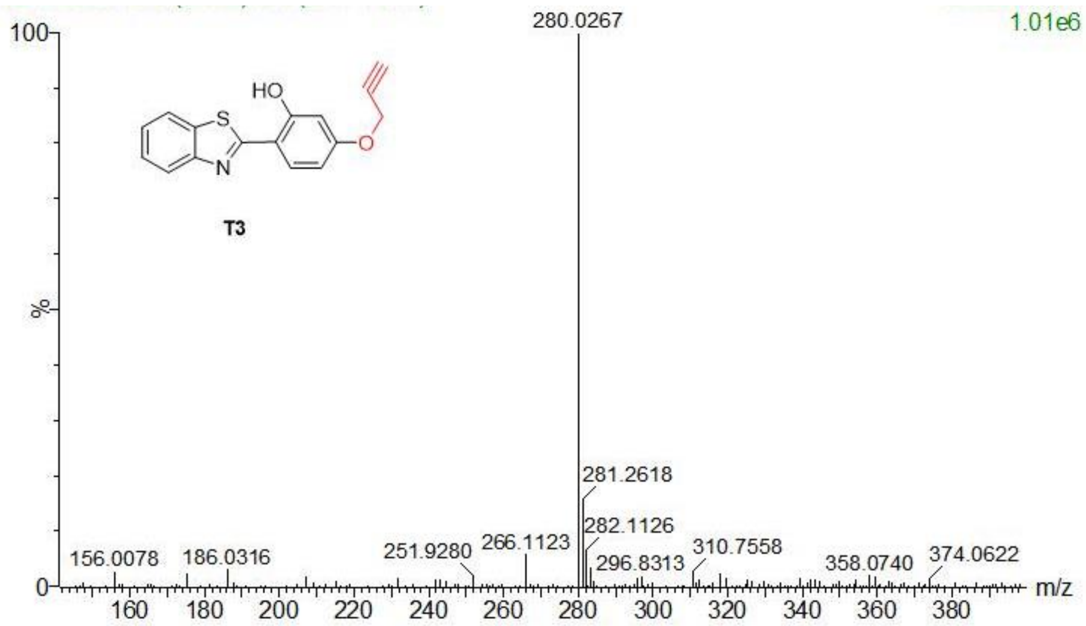

Figure S6. ^{1}H, ^{13}C NMR and MS of **T3**.

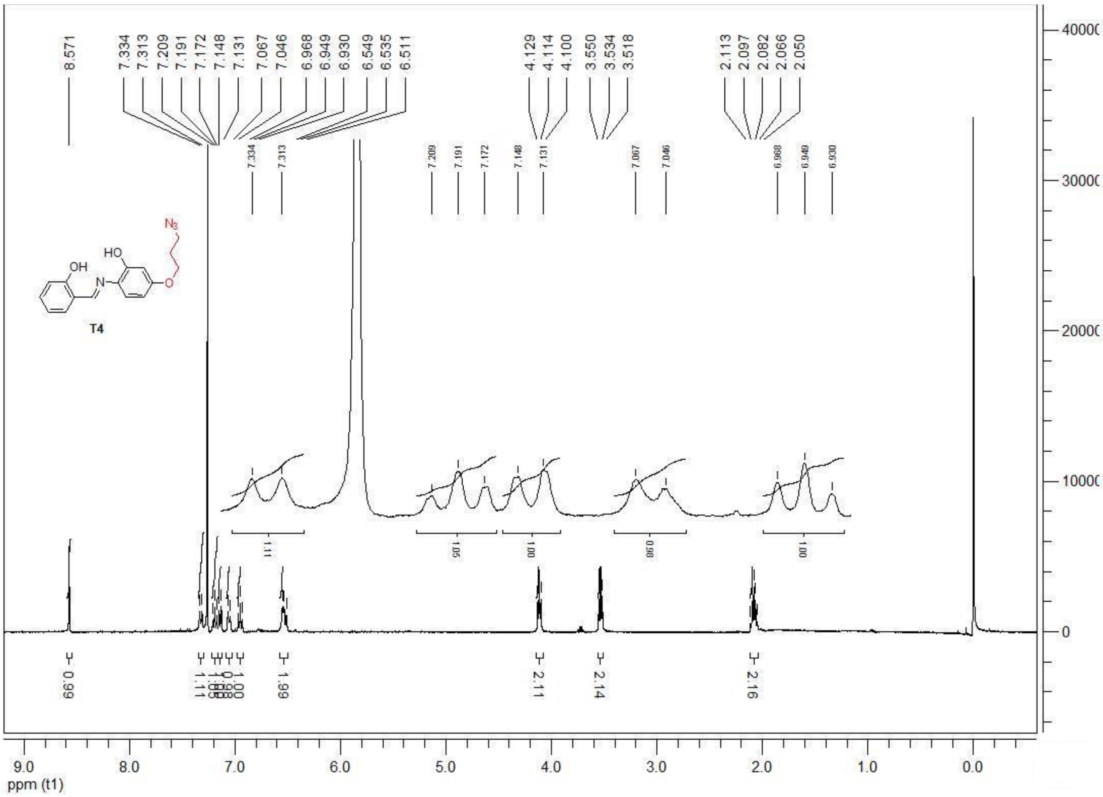
8.571
7.334
7.313
7.209
7.191
7.172
7.148
7.131
7.067
7.046
6.968
6.949
6.930
6.549
6.535
6.511
4.129
4.114
4.100
3.550
3.534
3.518
2.113
2.097
2.082
2.066
2.050
OH
HO
N
O
N_3
T4
0.99
1.99
2.11
2.14
2.16
ppm (t1)

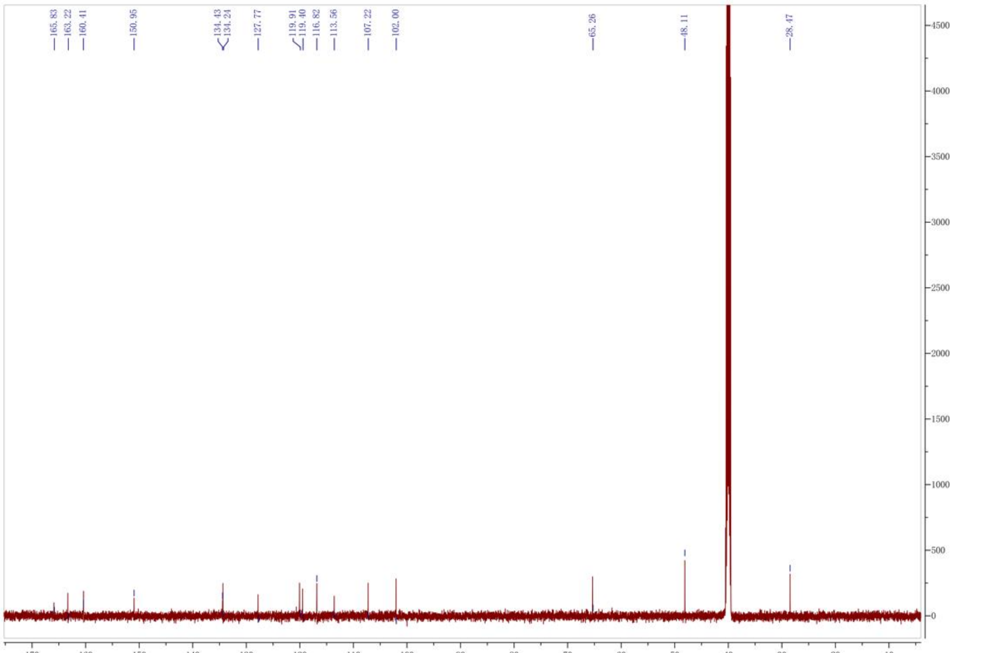
165.83
163.22
160.41
150.95
134.43
134.24
127.77
119.91
119.40
116.82
113.56
107.22
102.00
65.26
48.11
28.47
f1 (ppm)

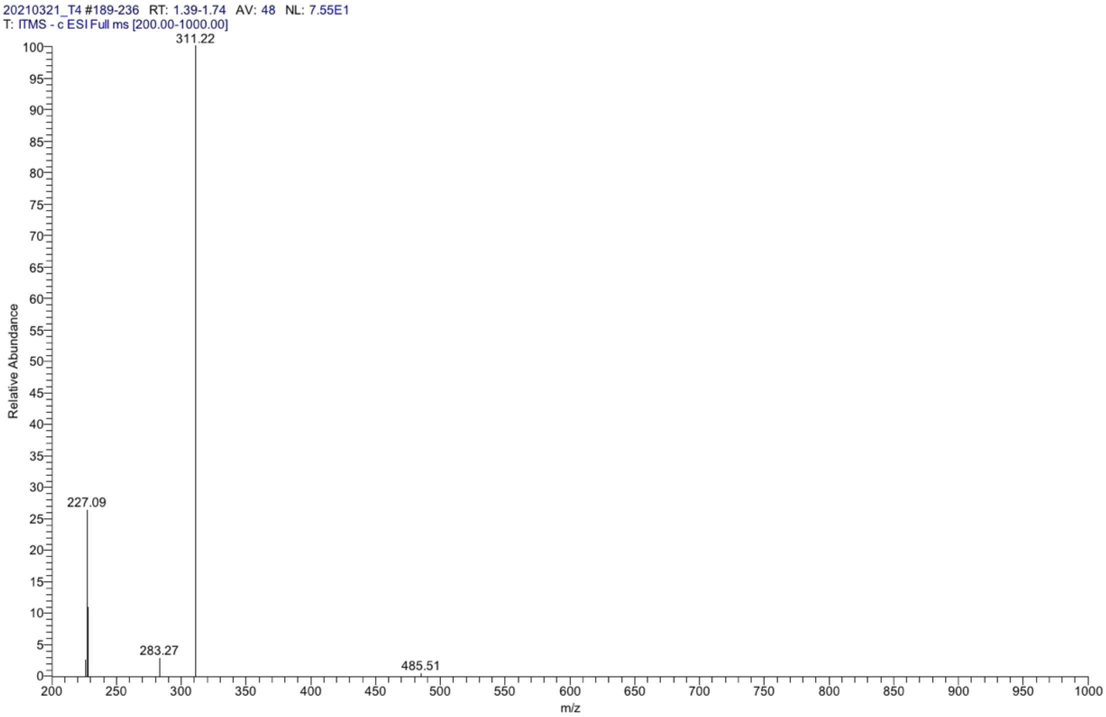

Figure S7. ^{1}H, ^{13}C NMR and MS of **T4**.

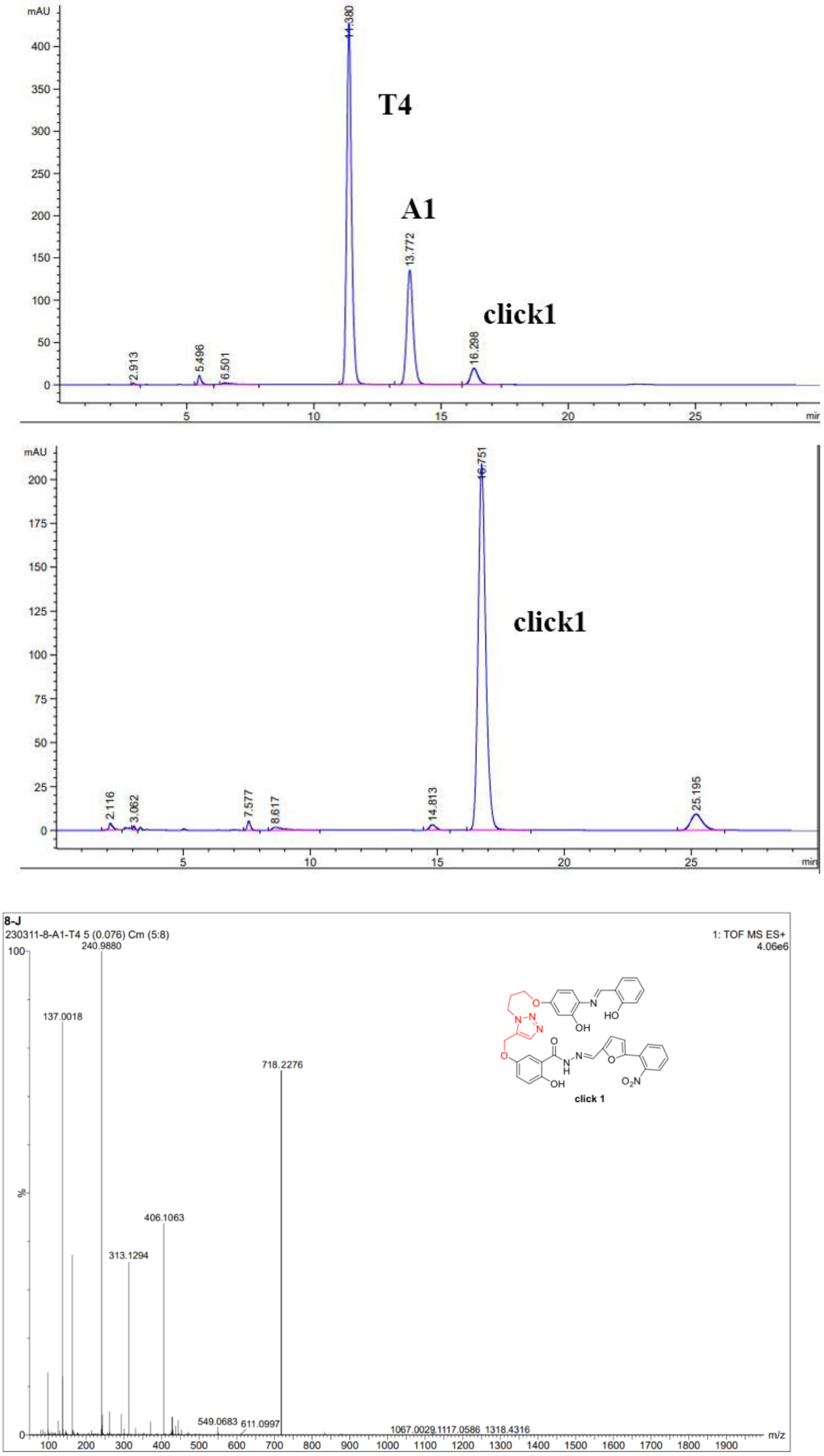

Figure S8. The HPLC-MS results for the click1 reaction.

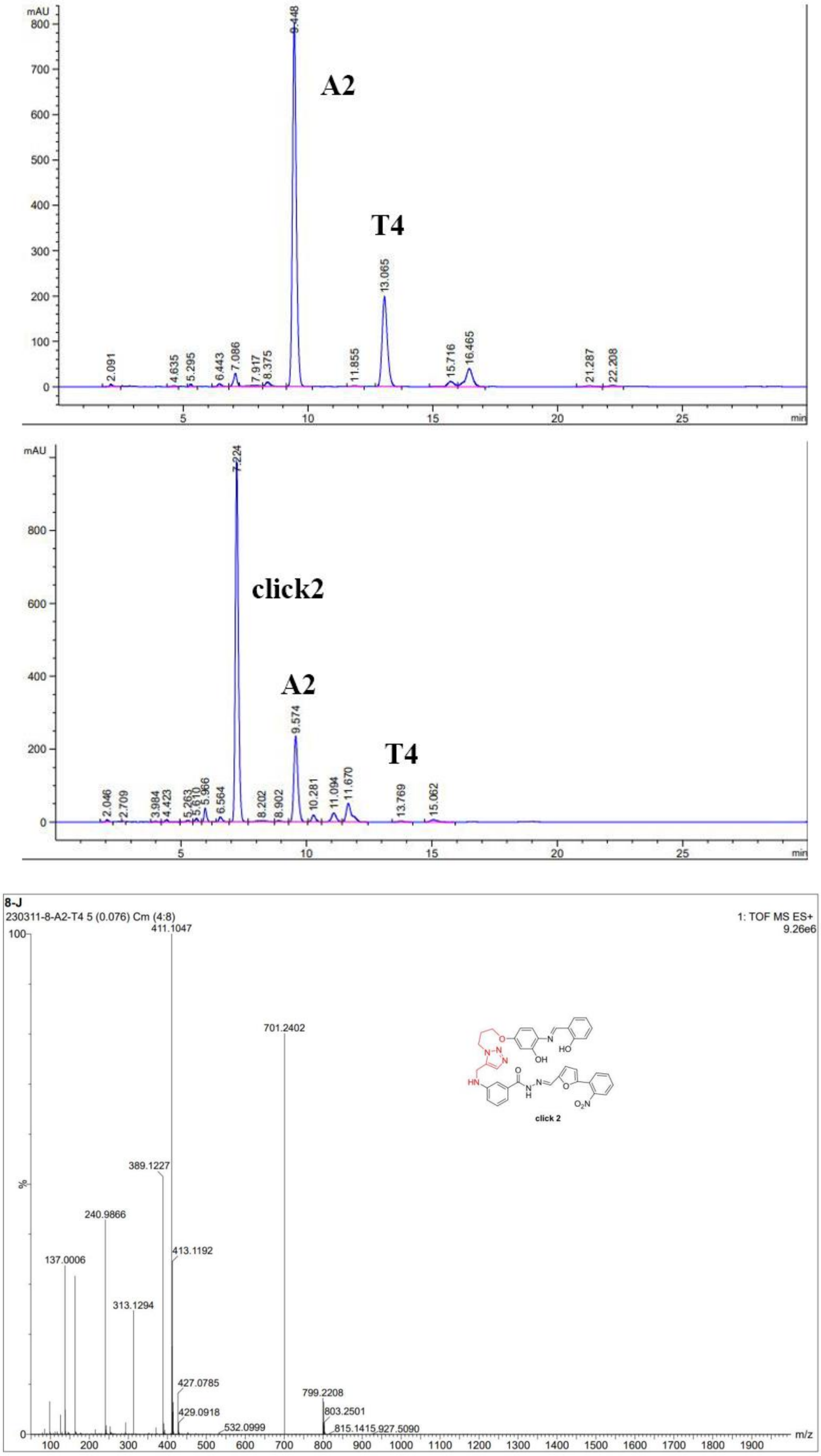

Figure S9. The HPLC-MS results for the click2 reaction.

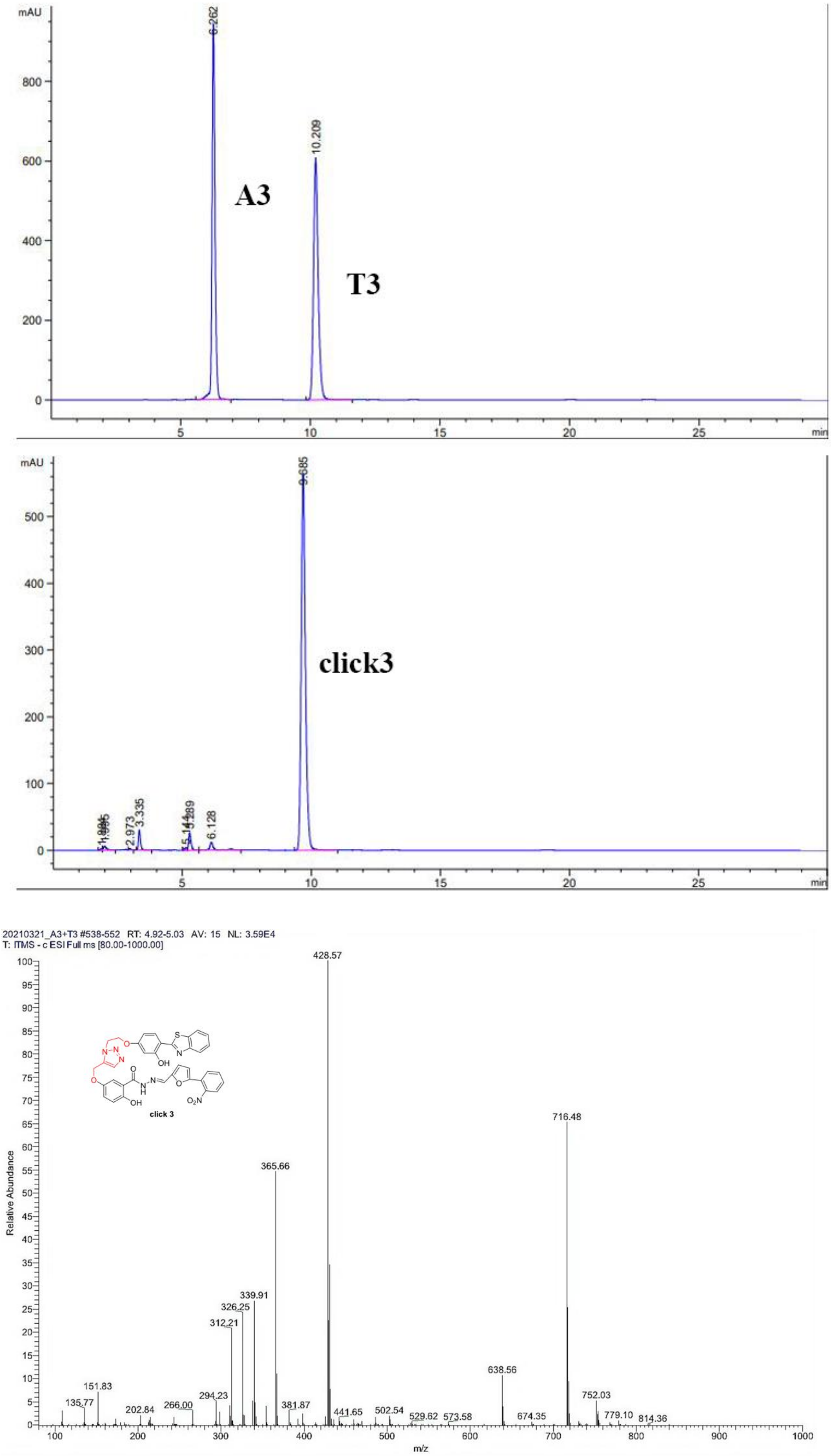

Figure S10. The HPLC-MS results for the click3 reaction.
